# Fast Gating for Raman Spectroscopy

**DOI:** 10.3390/s21082579

**Published:** 2021-04-07

**Authors:** Andrea Chiuri, Federico Angelini

**Affiliations:** Diagnostic and Metrology Laboratory, FSN-TECFIS-DIM Nuclear Fusion and Safety Technologies Department, ENEA Via Enrico Fermi 45, 00044 Frascati, Italy; andrea.chiuri@enea.it

**Keywords:** time-gating, Raman, ICCD, Kerr, SPAD

## Abstract

Fast gating in Raman spectroscopy is used to reject the fluorescence contribution from the sample and/or the substrate. Several techniques have been set up in the last few decades aiming either to enhance the Raman signal (CARS, SERS or Resonant Raman scattering) or to cancel out the fluorescence contribution (SERDS), and a number of reviews have already been published on these sub-topics. However, for many reasons it is sometimes necessary to reject fluorescence in traditional Raman spectroscopy, and in the last few decades a variety of papers dealt with this issue, which is still challenging due to the time scales at stake (down to picoseconds). Fast gating (<1 ns) in the time domain allows one to cut off part of the fluorescence signal and retrieve the best Raman signal, depending on the fluorescence lifetime of the sample and laser pulse duration. In particular, three different techniques have been developed to accomplish this task: optical Kerr cells, intensified Charge Coupling Devices and systems based on Single Photon Avalanche Photodiodes. The utility of time domain fast gating will be discussed, and In this work, the utility of time domain fast gating is discussed, as well as the performances of the mentioned techniques as reported in literature.

## 1. Introduction

Raman spectroscopy is a powerful tool for chemical analysis, providing both sensitive and selective measurements [1]. On the other hand, a drawback of this technique is given by the generally small cross-section, and the possible overimposed fluorescence signal (both from the sample and the substrate) that possibly covers the weak Raman signal. As observed in [2], fluorescence occurs concurrently only with Stokes Raman lines, since anti-Stokes Raman lines are blue-shifted with respect to the excitation radiation. However, under normal conditions, only Stokes lines are visible, or in any case, they are the most intense and hence the most used for chemical identification.

**Figure 1:**
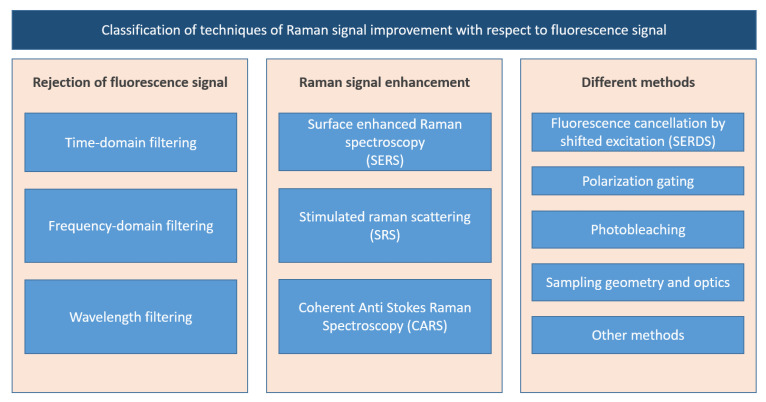
Graphic classification of methods for Raman enhancement regarding fluorescence signals.

To overcome this problem, a variety of techniques have been set over in the past few decades [3]. As summarized in that work, the possibilities range from suppressing fluorescence signal to enhancing the Raman signal, to other possibilities based on polarization or specific optical layouts. A graphic scheme of the classification is reported in Figure 1. Enhancement of Raman scattering can be obtained by coherent anti-Stokes Raman spectroscopy (CARS), surface enhanced Raman spectroscopy (SERS), Resonant Raman Scattering or stimulated Raman spectroscopy, while those aiming directly to eliminate the fluorescence contribution are shifted excitation Raman difference spectroscopy (SERDS), narrow spectral filtering, phase-sensitive detection and fast time gating. Extensive reviews on some of these subtopics can be found in [4,5,6,7,8]. However, no reviews have been published so far about the time-domain gating for Raman spectroscopy, a widely used technique for fluorescence suppression. In fact, most of the mentioned techniques require either sample preparation, or a specific wavelength selection for each compound under inspection. Moreover, sometimes further radiation is requested for the excitation of the energetic levels entering the Raman emission, causing photo-degradation of the sample.

The IEC 60825-1 international standard provides the guidelines to evaluate the maximum permitted exposition (MPE) to laser radiation according to a series of the beam parameters: energy, pulse duration, total exposure time, repetition rate, beam section, divergence and wavelength. Though a combination of single pulse energy and repetition rate can be found to maximize the Raman signal for a given exposure time in certain spectral regions [9], the problem of fluorescence is not bypassed, since fluorescence signal is increased as well, if UV radiation is employed. On the other hand, the IR window between 1500 and 1800 nm allows increasing the permitted dose, and in theory the Raman signal could be enhanced in a fluorescence-free scenario. However, the very small cross-section (100 times smaller than the 355 nm one), the performances of IR cameras and the low availability of lasers in this region make this choice currently hard to use, especially in biological samples where the strong water absorption could further weaken the Raman signal. Another important drawback is given by the very large Raman shift, in terms of wavelength, associated with Raman spectra in the IR, which obliges one to gather a very large spectral region, where optical efficiencies could be rather inhomogeneous: if 1540 nm excitation radiation is used, water vapor Stokes lines fall at about 3000 nm.

Classic Raman spectroscopy represents then, in theory, the most convenient technique for the reliable identification of the largest number of species with a minimal exposition of the sample. As mentioned, the main drawback is the small cross-section, and fluorescence may become a problem, especially if short wavelengths (250–400 nm) are used to increase the Raman cross-section.

In theory, the spectral range below 270 nm can be used as a relatively fluorescence-free signal, since most fluorescence spectra start from about 270 nm. However, in this region the absorption spectra may show sharp increases due to resonance, and no absorption below the resonance [10]. This causes nonlinearity in responses among different compounds, and limits the number of detectable species, since every compound may show resonance at a different wavelength. Hence, it cannot be suggested as a standard technique to overcome the fluorescence interference. Since the fluorescence spectra are usually much broader than Raman peaks, a solution could be to subtract the fluorescence as a baseline, provided that the signal is also collected at other wavelengths close to the Raman one. On the other hand, a narrow spectral filtering allows one to eliminate most of the fluorescence signal, which is emitted at different wavelengths. Nevertheless, because of the Poisson process of light measurement, the signal variance equals its average value: this means that the fluctuations associated with large signals may mask weaker signals overimposed on them.

Fortunately, other methods allow one to avoid fluorescence, as mentioned before. Another method employs temporal gating to exploit the slower timescales of fluorescence with respect to the Raman emission. Three techniques are currently used for fast gating.

The first is the use of a time-gated intensified CCD (ICCD), where the signal emitted from a photocatode is amplified only inside a short time interval and includes the light pulse (in general from a laser). This method has been proven as effective in rejecting fluorescence [11]. Nowadays, gating times as short as 200 ps are available on the market, so that a great part of the fluorescence echo can be cut off. However, 200 ps does not provide excellent fluorescence rejection for fluorophores with short decay times. In any case, ICCDs are very expensive instruments, and exclusively coupled to a CCD, which is not necessary the best detector in any application.

To reduce the cost and improve the integration capability of the detector, time-gated single photon avalanche diode (TG-SPAD)-based CMOS pixel circuits are a possible solution. These very fast detectors are able to detect single photons and allow gating times as short as 100 ps and less.

The third method employs an optical shutter (e.g., the Kerr cell, the Pockels cell or a photoacusitc cell) to allow light transmission only in a short time lapse. Among all these effect, a sharp change in the optical properties of a medium (crystalline or even amorphous) causes the birefringence of the medium which, if appropriately exploited, lets the medium become transmissive for a short time. The Kerr effect, in particular, can be driven by the laser light itself (optical Kerr effect) so that a single laser pulse may be used to excite the Raman spectrum and gate the detection, obtaining time gates as fast as the laser pulses. If a picosecond laser is employed, picosecond gating times can be easily reached. Nevertheless, this technique has the disadvantage of single-channel detection, which requires a scan of wavelengths to acquire a complete spectrum. In the following the advantages and drawbacks of each method will be discussed. In Section 2, some theoretical considerations about the performances achievable with fast-gated Raman detectors are presented. In Section 3, an overview of the gate based upon the optical Kerr cell is given, and theoretical calculations about the efficiency of this approach are discussed. Section 4 and Section 5 describe the setups employing single photon avalanche photodiodes and intensified CCDs, even coupled to a streak camera. A summary of the contents, and final remarks about the performances, pros and cons of each technique, is finally given in Section 6.

## 2. Fast Gating: Expected Performances

**Figure 2:**
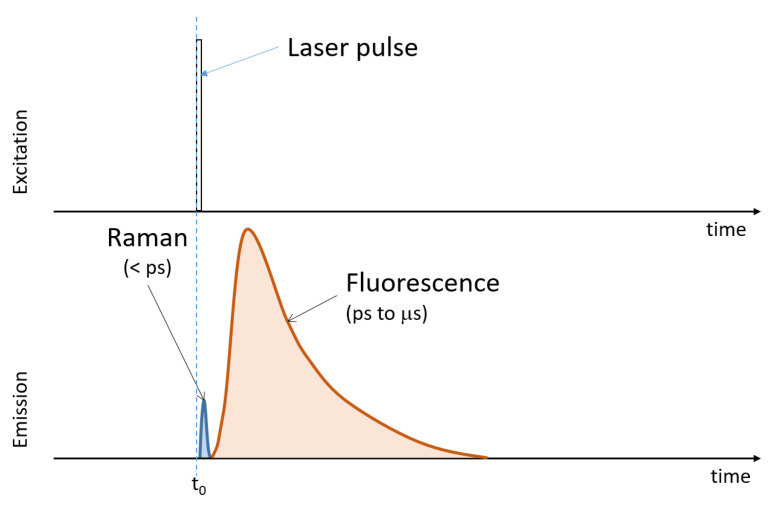
Temporal profile of the excitation laser pulse, emitted Raman scattering signal and emitted fluorescence.

A simple model can be set up to establish which part of a fluorescence signal can be cut off by a given time gate. The scheme of fluorescence emission is described through the Jablonsky diagrams. While the Raman signal can be assumed as instantaneous, fluorescence build-up time is typically of the order of picoseconds [2], as shown in Figure 2. During this period, called the internal conversion period, a series of non-radiative processes leads the electronic states to the lowest excited electronic level, and then the fluorescence consists of the return to the ground electronic state (although excited vibrational levels are possible), and the result is a series of photons of different energies, or under a macroscopic point of view, light of different wavelengths because of the many different transitions between excited vibrational states of the upper electronic level and the excited vibrational levels of the ground state.

This process of re-emission happens in times spanning from less than a nanosecond to hundreds of nanoseconds (the fluorescence lifetime $\tau_{f}$), and exponential decay is generally observed for fluorescence intensity for each fluorophore. Unfortunately, the fluorescence signal may overpass the Raman one by several orders of magnitude, and its intrinsic fluctuations significantly lower the signal to noise ratio achievable in Raman detection.

**Figure 3:**
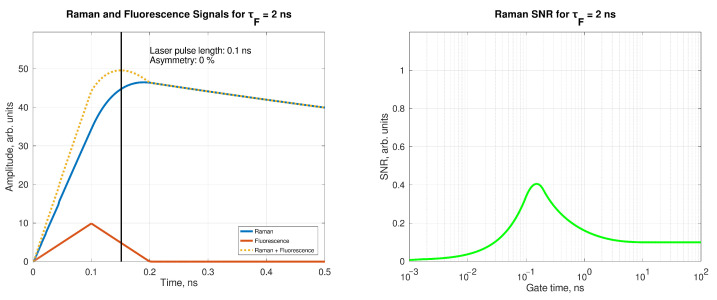
The Raman, fluorescence and total (Raman + fluorescence) signals as a function of time (**left**) and the Raman signal-to-noise ratio (RSN) as a function of the gating time. The black line in the left plot represents the gating time at which the best RSN is obtained, i.e., the abscissa of the maximum in the (**right**) plot.

**Figure 4:**
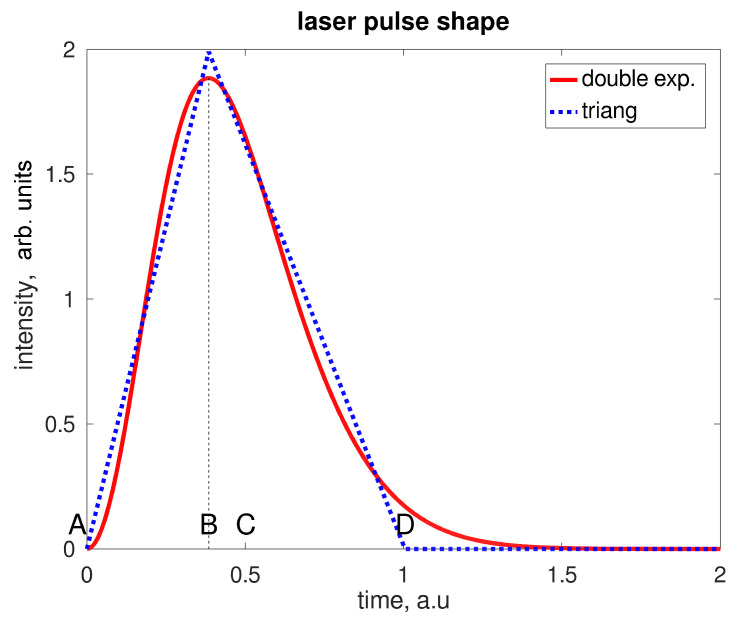
Approximation of a double exponential laser pulse with a triangular function having an equal area. The parameters of the triangular function are determined so that the modal value is the same as that of the double exponential, and to ensure an even ratio for the areas to the left and to the right of the modal value (indicated by the dotted line). Point A represents the beginning of the pulse; B is the time where the peak value is reached; C is the middle point between A and D; and D is the end point of the triangular pulse.

When a laser pulse hits a fluorophore, the convolution between the laser intensity and the fluorescence decay determines the temporal shape of the signal re-emitted by the sample. In Figure 3, the total Raman (blue) and the fluorescence (red) signals are plotted versus time together with the total signal (yellow), for 0.1 ns full width half maximum (FWHM) pulse width. The laser pulse intensity can be generally described as a double exponential [12,13], but by following [14] it can be approximated by a triangular function of time, as shown in Figure 4, for better control of the effects of function parameters on the signal properties (area, symmetry, duration).

In this study the fluorescence signal is imposed to be 100 times stronger than the Raman one; this latter is obtained as the difference between the total signal and the fluorescence signal (the two measurable quantities), and a proxy of the performances of the rejection system can be calculated as the $Raman/\sqrt{Total}$. This quantity can be defined as Raman signal-to-noise ratio (RSN)m and it depends on the gating time. In the best case, Raman is completely detected and fluorescence is completely rejected; this value will equal 1. In the worst case where all the Raman and fluorescence signals are detected it will equal $1/\sqrt{101}=0.0995$.

In fact, if the signal is integrated over a very short time (<<FWHM), neither Raman nor fluorescence signals will be detected, giving a very small RSN. On the other hand, if the gating time is very long (≈μs), all the fluorescence signal will be detected, and the error associated with the measurement will be ten times the Raman signal itself. Between these two extreme choices, a gating time close to the laser pulse length may be imposed to stop the acquisition, and the Raman signal will become more visible, according to the laser pulse length and the fluorescence lifetime. It is intuitive that the longer the fluorescence decay, the easiest the fluorescence cutoff. However, a complete rejection of fluorescence is not possible, unless the laser pulses are shorter than ≈ps; in this case, however, the spectral broadening associated with short pulses makes the spectral resolution too low for effective Raman detection.

Additionally, the shape of the laser pulse could influence the ability of rejection: If the peak is very close to the beginning (a realistic behavior) the rejection ability is expected to be better than for a symmetric pulse. An asymmetry factor ≈ may be defined as the ratio between the distance between the beginning of the signal and the peak and the middle point of the signal. In terms of the points defined in Figure 4, $\alpha =\bar{CB}⁄\bar{AC}$.

A symmetric pulse has a null asymmetry factor. However, the effects of the pulse shape on the performances reached by the time-gated spectroscopy are not so significant. The model calculates the best RSN according to laser pulse length FWHM, fluorescence lifetime $\tau_{f}$ and the asymmetry factor of the laser pulse. As a first result, it can be inferred that the best RSN is always achieved for gating times about 1.7 times the laser FWHM, with only a marginal impact due to the asymmetry of the pulse. Only shapes where B is found to the left of C are considered; results are shown in Table 1.

**Figure 5:**
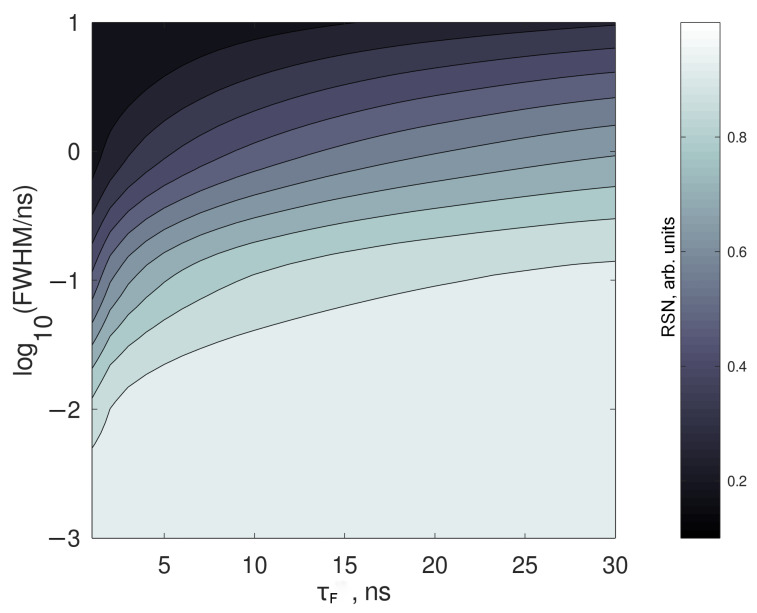
The best RSN achievable in a time-gated Raman system when a 100-times stronger fluorescence signal is overimposed to Raman signal, as a function of the fluorescence decay time and the laser full width half maximum pulse length. The gating time is always about 1.7× FWHM. Pulse asymmetries do not significantly change the results.

Another result is shown in Figure 5, where the best RSN achievable is plotted against the fluorescence lifetime and the FWHM. It is evident that laser pulses longer than 100 ps never allow a complete fluorescence cutoff, especially for short-lived fluorophores.

From these results the advantages of an optical Kerr cell are evident, as the gating time can be kept as short as the laser pulse: employing picosecond lasers this would enable very high RSNs. Some details on the proposed methods will be described in the following sections.

## 3. Optical Kerr Gate

Some materials are subject to changes in optical properties when subjected to an electric field [15,16]. In fact, electromagnetic forces may alter the microphysical properties of atoms and molecules of the material (position, orientation or shape). This is reflected in changes in the refractive index, for example, after the application of a low-frequency electric field. If the material is anisotropic, the field applied modifies its refractive indices and its behavior in polarized light.

The dependence of the refractive index on the applied electric field takes one of two forms:

- The refractive index changes in proportion to the applied electric field, in which case the effect is known as the linear electro-optic effect or the Pockels effect. The linear electro-optical effect can be described in terms of a nonlinear polarization given by:
  (1) $$P_{i}\left(\omega \right)=2\sum_{jk}\chi_{ijk}^{\left(2\right)}\left(\omega +0\right)E_{j}\left(\omega \right)E_{k}\left(0\right)$$
  since the linear electro-optic effect can be described by a second-order nonlinear susceptibility [15], it follows that a linear electro-optic effect can occur only for materials that are non-centrosymmetric (i.e., they do not display inversion symmetry). In fact, for centrosymmetric ones $\chi^{\left(2\right)}=0$ and $\chi^{\left(3\right)}\neq 0$; for non-centrosymmetric, $\chi^{\left(2\right)}\neq 0$ and $\chi^{\left(3\right)}\neq 0$ (with $chi^{\left(2\right)}\;chi^{\left(3\right)}$). Furthermore, in this case a refractive index ellipsoid should be defined; hence, the material cannot be amorphous (i.e., with the same refractive index along each direction) and only crystals could be considered. Since in this case there is only a linear variation of the refractive index, the centrosymmetric crystals do not display this effect because of their inversion symmetry. Nowadays, very fast Pockels cells are produced by some manufacturers, who declare gating times as short as 150 ps, with window diameters up to 6 mm. These devices could represent a promising tool for fast Raman gating, though no research articles have been published so far on this topic.
- The refractive index changes in proportion to the square of the applied electric field, in which case the effect is known as the quadratic electro-optic effect or the Kerr effect. This occurs in centrosymmetric materials (i.e., they display inversion symmetry), where the lowest-order change in the refractive index depends quadratically on the strength of the applied field. It can be described in terms of a nonlinear polarization given by:
  (2) $$P_{i}\left(\omega \right)=3\sum_{jkl}\chi_{ijkl}^{\left(3\right)}\left(\omega +0+0\right)E_{j}\left(\omega \right)E_{k}\left(0\right)E_{l}\left(0\right)$$
  Due to dependence by $\chi^{\left(3\right)}$, the non-linearity can be induced by placing the medium inside DC or AC electric fields (e.g., a sort of capacitor can be used); otherwise, the electric field can be generated by a strong radiation (e.g., a laser beam). The latter, usually known as the optical Kerr effect, allows one to improve the performances, and lower gating windows (e.g., ps) could be achieved.

In any case, refractive index variations induced in this way are usually very small. Nevertheless, they can induce significant effects if the wave propagates over distances much larger than its wavelength, so that small phase changes may induce measurable total phase shifts. As an example, for a refractive index change of $10^{-5}$, an optical wave will experience an additional shift of 2π after $10^{5}$ wavelengths. These materials can be used to realize electrically controlled optical devices—for example:

- Lenses, where the the focal length can be varied changing the refractive index;
- Prisms, where the beam deviation is controllable by changing the refractive index, becoming devices capable of scanning;
- Phase modulators, where light transmitted undergoes a controllable phase shift;
- Wave retarders, where anisotropic crystals may become able to change the polarization properties of the beam crossing the medium;
- Optical switches, where wave retarders between two crossed polarizers cause transmission of the whole device to be dependent on the phase shift introduced.

Based on the idea expressed with the last example, the Kerr effect was employed for time-resolved Raman spectroscopy (TRRS) [3,17,18,19,20,21].

One of the first experiments [17] was based on a Kerr gate with a resolution of few picoseconds at a 102 Hz repetition rate and allowed the experimenters to detect light with a spectrometer and CCD combination. These systems achieved a good suppression of the background from fluorophore with a lifetime of about 2 ns.

As previously described, in a Kerr cell the non-linearity of an optical medium is exploited to introduce an optical phase delay and to rotate the polarization of the impinging beam. In this work the non-linearity induced by a laser beam will be considered since this approach paves the way for the use of the same laser pulse to stimulate the Raman signal and to activate the cell (i.e., optical Kerr effect). In this way, the fluorescence, which occurs after a few picoseconds, can be rejected with very high efficiency at the end of the laser pulse.

Furthermore, the optical pumping allows one to use any pulse repetition rate (PRR) to integrate the signal, since no electronic trigger is required during the acquisition phase. On the other hand, the use of very high PRR and very short pulse lasers (up to picoseconds) should be preferred, in order to increase the signal since the single pulse energy is usually not very high.

### 3.1. Nonlinear Refractive Index

The Kerr effect relies on the non-linearity induced into a medium. Let us introduce the refractive index as $n=n_{0}+\Delta n$, where $n_{0}$ is the linear term, while $\Delta n=n_{2}\cdot E^{2}$ is a function of the incident field E (i.e., a laser) and the nonlinear refractive index $n_{2}$ [15]. By exploiting these properties, it is possible to introduce a phase ϕ between two orthogonal components of a polarized beam which can be expressed as follows:

(3) $$\phi =\frac{2\pi \Delta n}{\lambda }L=\frac{2\pi n_{2}}{\lambda }LI$$

where *L*: thickness of the nonlinear medium; λ: wavelength of the gated pulse—that is, the Raman signal to be rotated by the Kerr medium, as will be described in the next paragraphs; *I*: power density of the pulse used to activate the gate (i.e., the nonlinearity in the crystal). Let us introduce the definition of the power density $I=\frac{E}{T\pi {\left(\frac{d}{2}\right)}^{2}}$, where *E*: pulse energy; *T*: pulse duration; *d*: laser beam diameter within the nonlinear medium. This leads to a more useful expression for the phase ϕ:

(4) $$\phi =\frac{8LEn_{2}}{\lambda Td^{2}}$$

By suitably varying the parameters reported in Equation (4) it is possible to create a half-wave plate (i.e., ϕ=π). In this case, if the crystal is rotated by an angle θ, the polarization of the emerging beam will be rotated by an angle by 2·θ. It is worth noting that the active medium will act as an half-wave plate only for one value of λ, once given the other parameters. For this reason, in Section 3.3 we will define a transfer function characterizing the Kerr cell efficiency at different wavelengths. Since the parameters included in Equation (4) represent physical properties with intrinsic uncertainty, they cause a variety of phase ϕ; hence, they will be considered in order to characterize the efficiency of a real setup.

### 3.2. Experimental Implementation

**Figure 6:**
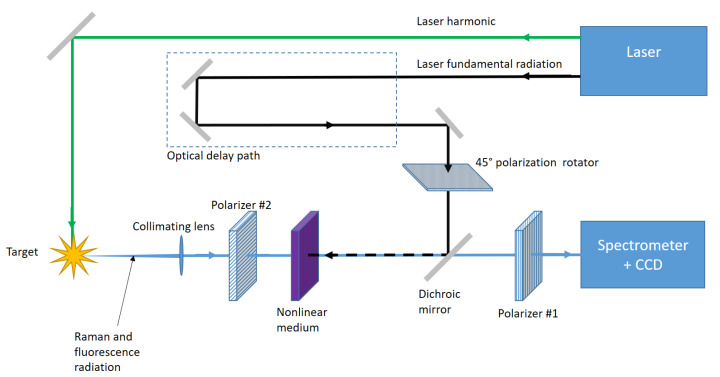
Experimental setup of a Kerr cell optically activated exploiting the properties of a nonlinear crystal.

Let us now introduce the experimental scheme reported in Figure 6 which could be exploited to generate an optical Kerr gate devoted to the TRRS.

The fundamental optical elements are [15,16]:

- The polarizer #1 (e.g., a polarizing beam splitter—PBS).
- The polarizer #2.
- The nonlinear medium.
- A laser able to emit at two different wavelengths, the first to excite the target (i.e., Raman Signal) and the second to induce the Kerr effect.

The Raman signal (blue in Figure 6) and the fluorescence impinge on the first polarizer and the emerging field oscillates along a well-defined plane. The pumping pulse (black in Figure 6), which induces the necessary birefringence within the active medium (i.e., the crystal), should be suitably synchronized with the Raman radiation. This is usually accomplished by a simple optical variable delay line obtained with mobile mirrors. Since the Kerr effect relies on high energy density, the active medium is usually pumped by exploiting the laser fundamental harmonic, while lower wavelengths are necessary to obtain a higher Raman signal.

The active medium should act as a half-wave plate (i.e., λ/2) at $45^{\circ }$ only during the pulse duration of the pumping laser; hence, the polarization of the radiation passing through the crystal in this time interval is rotated by $90^{\circ }$. The second polarizer, orthogonal with respect to the first one, allows one to select the Raman signal which was suitably rotated and to discard the undesired fluorescence.

In order to obtain a half-wave plate at $45^{\circ }$, you could rotate the crystal or you can follow a different approach, reported in Figure 6, where the gating pulse passes through a polarizer at $45^{\circ }$ or is rotated by a half-wave plate at $45^{\circ }$. In this case the birefringence generated in the nonlinear crystal allows one to realize a result equivalent to that achievable by rotating the active medium.

Several schemes of optical Kerr gate devoted to the TRRS were reported in literature [3,17,18,19,20]. The choice of a particular setup could be based on reasons related to the cost, technical characteristics (i.e., weight, dimensions, power) or just the availability on the market. More precisely:

- Most setups involve a picosecond laser, such as diodes @ 1064 nm [19] or solid state systems at 800 nm (e.g., Ti:sapphire laser) [17,18,22].
- Even though the same laser is usually exploited to obtain the Kerr effect and for the Raman signal, for reasons of costs and synchronization (in this case, doubled-frequency radiation should be generated), there is not a theoretical constraint on the use of different wavelength combinations. However, further assessments should be considered in order to get as many Raman signals as possible. A low absorbance of the active medium in the chosen range, and a Raman pump close to the UV to increase the cross-section would be suitable. A solution commercially available could involve a gating pulse at 1064 nm and a Raman pump at 532 nm, i.e., a gated pulse close to 532 nm or both the gating and gated pulses at 532 nm. In the present paper we will focus on this easier configuration.
- The active medium could be a liquid (CS${}_{2}$) [17,18,23,24] or a solid (TiO${}_{2}$, CdS, ZnO, bismuth glass) [19,20]. Several devices were optimized in order to exploit the TiO${}_{2}$ by varying the size and the configuration of the active medium, which could be homogenous or variously dispersed. Nevertheless, the choice of the active medium is based on the value of the nonlinear refractive index which should be as high as possible.

In this work, some simulations have been performed considering TiO${}_{2}$. This could be a good candidate for a setup devoted to the TRRS, since it has low absorbance in the visible range [25,26,27,28,29,30], which increases in the UV range, and it is less affected by problems than other media, e.g., low two-photon absorbance, because of the higher energy gap. In fact, multi-photon absorption of the gate pulse in the Kerr medium can cause a strong background emission [31].

### 3.3. Kerr Gate Efficiency

In this section, we report a performance analysis of the setup from Figure 6 by considering not only ideal configurations but even real conditions achievable with commercial tools and optical elements (e.g., laser, active medium and PBS).

The phase factor reported in Equation (4) should be as close as possible to π in order to obtain an optimal gate. This condition can be achieved only for one λ by suitably adapting the involved parameters which are characterized by intrinsic uncertainty.

The total efficiency of the Kerr gate can be estimated by considering the Jones matrix formalism [32] for nonlinear optical elements (LR(ϕ)) introducing a general phase factor ϕ between two orthogonal polarizations (i.e., horizontal and vertical), which is rotated by angles θ (R(θ) and R(−θ)):

(5) $$LR\left(\phi ,\theta \right)=R\left(-\theta \right)LR\left(\phi \right)R\left(\theta \right)=\left(\begin{array}{cc} \cos(-\theta ) & \sin(-\theta )\\ -\sin(-\theta ) & \cos(-\theta ) \end{array}\right)\cdot \left(\begin{array}{cc} 1 & 0\\ 0 & e^{-i\phi } \end{array}\right)\cdot \left(\begin{array}{cc} \cos(\theta ) & \sin(\theta )\\ -\sin(\theta ) & \cos(\theta ) \end{array}\right)$$

Let us consider the usual orthogonal base for the polarization state composed by the vector $\left(\begin{array}{c} 1\\ 0 \end{array}\right)$ for the horizontal state and $\left(\begin{array}{c} 0\\ 1 \end{array}\right)$ for the vertical one. The PBS#1 selects only one polarization state; here the state $\left(\begin{array}{c} 1\\ 0 \end{array}\right)$ is considered but this model is valid even with a different choice. Hence, the output of the system composed by PBS#1 and active medium is given by the following product:

(6) $$\left(\begin{array}{c} out_{H}\\ out_{V} \end{array}\right)=LR\left(\phi ,\theta \right)\left(\begin{array}{c} 1\\ 0 \end{array}\right)$$

and $|out_{V}|$ is related to the the probability to have an output field vertically polarized ($prob_{V}$), i.e., the only state which can pass the PBS#2.

It follows that the total efficiency can be given as the product:

(7) $$\eta_{tot}=prob_{V}\cdot \eta_{PBS}\cdot \eta_{AM}\cdot \eta_{rot}$$

where $\eta_{AM}$ depends on the transmittance of the active medium (e.g., $\eta_{AM}=0.85$ for the TiO${}_{2}$ at 532 nm [29,30]), $\eta_{PBS}$ quantifies the efficiency of the PBS#2 (commercial PBS achieves an extinction ratio of 1:1000; hence $\eta_{PBS}=0.999$) and $\eta_{rot}$ represents the efficiency of the rotation at $45^{\circ }$ of the active medium or the errors which can be made in equivalent experimental configurations.

This simple model can be used to estimate the efficiency for the whole Raman signal passing through the crystal, since the peak is narrow and the same properties (e.g., the transmittance of the active medium, the transmittance of the PBS, $n_{2}$ and *n* are roughly constant) can be considered for all the wavelengths.

### 3.4. Setup Design

**Figure 7:**
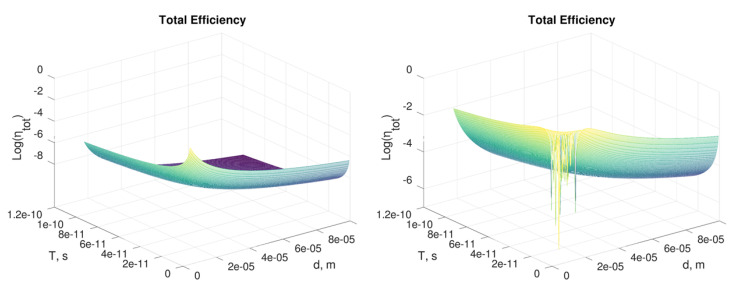
**Left**: $Log(\eta_{tot})$ vs. E,T; **right**: $Log(\eta_{tot})$ vs. E,d.

Let us consider the results obtained in Equation (7) and the values for the parameters reported in Equation (2) based on those of commercial lasers. The other terms are: *d* = 1 mm (not focused beam), *L* = 1 mm, λ = 532 nm and TiO${}_{2}$ as active medium. In this case a low efficiency (i.e., <0.001%) is achieved even for short T (1 ps) and high E (500 nJ) (see Figure 7a). Better performances can be obtained by varying d within the diffraction limit (see Figure 7b), even though, in this case, it should be difficult to guarantee optimal overlap between the gating and the gated pulses. Different active media or other configurations of TiO${}_{2}$ [17,18,19,20,23,24,25,26,27,28,29,30] would bring about results similar to those reported in this section.

**Figure 8:**
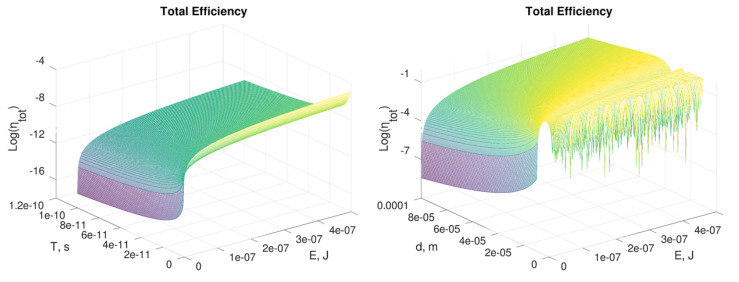
**Left**: $Log(\eta_{tot})$ vs. T,d (E = 1 nJ); **right**: $Log(\eta_{tot})$ vs. T,d (E = 100 nJ).

We can conclude that d=10μm is a feasible value, although commercial beam shapers could guarantee a lower size, up to 1μm. The other relevant parameter, E, should be as high as possible and depends on the peak power of the particular system. Nevertheless, high average and peak power of laser radiation (e.g., MW) cannot be considered, because that can induce sample damage and nonlinear processes. Figure 8 demonstrates that by varying only E from 1 to 100 nJ, the same setup could reach the maximum achievable efficiency.

By exploiting Equation (4), it is possible to estimate the pulse energy needed to obtain a phase ϕ=π as $E=\pi d^{2}\lambda T/8Ln_{2}$. In Table 2 we report several values of E for different configurations (i.e., pulse diameter and pulse duration) of a Kerr cell based on TiO${}_{2}$ as active medium.

Several kinds of commercial lasers are available in the range T=[1,100] ps characterized by technical specifications suitable for a TRRS. It is worth noting that, in these cases, d should be very low. A few examples, without citing specific laser manufacturers or suppliers, follow.

- PRR = 100 MHz, T ≈45ps, W > 0.1 W, E > 1 nJ—$\eta_{tot}=1.8\%$ with d = 1μm and <<1% with d = 10μm.
- PRR = 40 MHz, T < 9 ps, W > 0.06 W, E > 1.5 nJ—$\eta_{tot}=68.2\%$ with d = 1μm and 0.01% with d = 10μm.
- PRR = 1–80 MHz, T = 88–96 ps, W = 0.0063–0.34 W, E = 4.3–6.3 nJ—$\eta_{tot}$ = 9–15% with d = 1μm and <<1% with d = 10μm.
- PRR = 80 MHz, T < 3 ps, W > 0.7 W, E > 8.5 nJ—$\eta_{tot}=\;\approx$3% even with d = 10μm.

A different approach reported in [19] involved a more expensive laser characterized by 4 ps pulse duration (sufficient for a Raman bandwidth of 2–3 cm${}^{-1}$ @532 nm), average power 0.5 mW and PRR 2 MHz; it follows that E=250 nJ. The reported total efficiency for this system is 10%, which could be roughly obtained with d≈35μm.

In the literature, a further solution was proposed by exploiting solid state lasers (e.g., Ti:Sa) at λ≈800 nm with CS${}_{2}$ ($n_{2}=3.1\times 10^{-18}$ m${}^{2}$/W) as active medium and T<<10 ps [18]. Such a device, however, is at the moment much more expensive than the others already described.

We should also mention systems based on commercial fs lasers [22,33,34,35] allowing one to achieve time resolution of ≈100 fs, but with limitations due to the spectral broadening (i.e., spectral resolution is too low for Raman detection) and experimental setups designed for ultrafast, or more generally, pump-probe, spectroscopy [36,37]. The latter represent valuable possibilities to be considered, but they involve high costs and complex optical setups. It is worth noting that this work is focused on a particular application of the Kerr shutters, that is, the TRRS. Hence, issues related to the response time of the involved medium will be not considered because the pulse duration cannot be lowered over the picosecond limit, as already discussed.

Another limit in designing efficient optical Kerr gating systems is represented by the availability of lasers and optical elements. The laser, in fact, should satisfy not trivial technical requirements in terms of PRR and average power, or single pulse energy. Deviations from ideal behavior could be related, for instance, to spectral broadening; inconsistent pulse duration; or PRR, anisotropy and size imperfections of the crystal—imperfect pulse shape.

It is now possible to point out several remarks, assessments and useful considerations for a high-performance setup:

- The efficiency of the setups reported in literature is roughly 10–15% [17,18,19,22].
- A setup devoted to TRRS should be based on a gated pulse of short wavelength in order to increase the cross-section.
- A low PRR should be preferred in order to obtain high pulse energy.
- Increasing the length of the active medium *L* does not lead to exceptional efficiency improvements: In fact, increasing the size could ease the presence of local inhomogeneities, higher absorbance, lower signal strength and problems related to the interfaces between the different slices. The latter happens when several layers of active medium are placed in series.
- Should the laser power exceed 300 mW, many setups are based on gating pulses generated with a laser at 1064 or 808 nm so that it is easier to find systems with the necessary technical features.
- Working with a large section beam, i.e., d≈ mm, short pulses are needed: T<<100ps. In this case the beam homogeneity represents a relevant issue. A solution could be represented by commercial beam shapers, able to make the beam energy density as constant as possible, e.g., “top hat beam shaping lenses” in order to reach values close to the diffraction limit [38].

### 3.5. Uncertainties and Their Consequences in Real Systems

Let us now describe how the deviations from the ideal behavior of the optical elements and tools can affect the total efficiency. Indeed, the phase factor cannot be always given predicted with absolute confidence, because of random fluctuations of the pulse energy and imperfections of the active medium. By deriving Equation (3), it is possible to obtain the expression of the fluctuations on ϕ in order to assess the more relevant term.

(8) $$\Delta \left(\phi \right)=\frac{2\pi LI}{\lambda }\Delta n_{2}+\frac{2\pi n_{2}I}{\lambda }\Delta L+\frac{2\pi n_{2}L}{\lambda }\Delta I+\frac{2\pi n_{2}LI}{\lambda^{2}}\Delta \lambda$$

In order to simulate a real system, realistic values were introduced into Equation (8) with their associated errors, and they are listed in Table 3 (a conservative approach was adopted).

A major contribution comes from the term $\frac{2\pi n_{2}L}{\lambda }\Delta I$, which depends on the beam variability, i.e., spatial and pulse-to-pulse variability. It is worth noting that in Equation (8) only *I* is considered, since $\phi =\frac{2\pi n_{2}}{\lambda }LI=\frac{8n_{2}LW}{\lambda d^{2}TPRR}$ and all the possible sources of error should be considered by introducing W, T and PRR.

A further analysis can be performed by simulating the effects of independent fluctuations of the parameters just discussed by exploiting a Monte Carlo model. Random variations were introduced for each parameter and the total efficiency was estimated. The probability distribution around the mean value were considered to be Gaussian with standard deviations equal to those reported in Table 3. The *i*-th term is given by: $X_{i}=X_{i}+randn\cdot \Delta X_{i}$ at each iteration (where *randn* is a random number with normal distribution with expected value = 0 and variance = 1). The values reported in Table 3 were used for each $X_{i}$ and $\Delta X_{i}$ with the only exception of λ; in this case a broader fluctuation was considered, i.e., $6\times 10^{-8}$, in order verify the behavior of the system over a wider range. For the rotation angle $\theta =45^{\circ }$, the maximum variation $\Delta \theta =0.5^{\circ }$ was considered.

**Figure 9:**
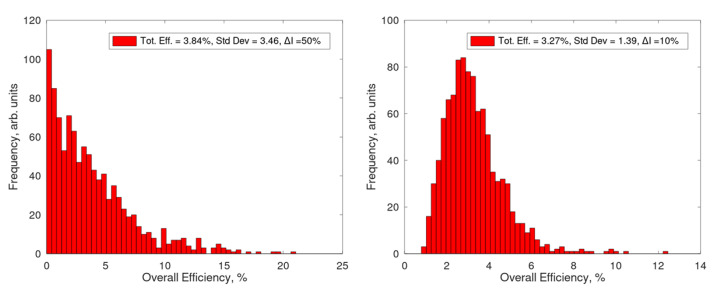
Total efficiency distribution obtained by the Monte Carlo simulation. Maximum fluctuation for ΔI: 50% (**left**); 10% (**right**).

The results, reported in Figure 9, demonstrate that the fluctuations are relevant and mainly due to the term ΔI; indeed, by considering a lower value (e.g., ΔI=10%) the standard deviation lowers from 90% to 43%.

**Figure 10:**
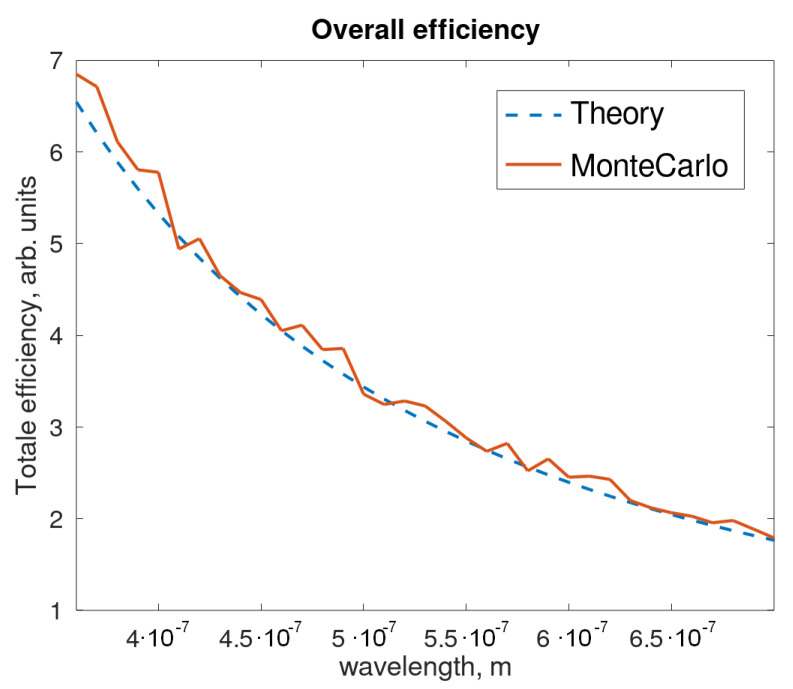
Simulation of total efficiency which could be obtained with a Kerr shutter characterized by the parameters reported in Table 3.

In Figure 10 a transfer function is shown in order to demonstrate the effects of random independent fluctuations (i.e., a Monte Carlo simulation with 100 iterations for each wavelength) of each parameter on the Kerr Gate. This was done by exploiting the values reported in Table 3.

### 3.6. Ideal and Real Gate

To perform Raman spectroscopy, however, a range of different wavelengths should be able to pass through the Kerr cell, and we have seen so far that each choice of the parameters can only allow a perfect gate for a precise wavelength. In order to estimate the performances of a cell over a broad range of wavelengths, a transfer function can be defined from the phase shift at each wavelength. As is intuitive, fluctuations of the parameters will alter this curve from the theoretical expectations.

The transfer function of an ideal setup represents an interesting issue to be explored and characterized for a wide range of wavelengths of the gated pulse. Precisely, a Kerr gate can be considered as ideal when it can induce a phase ϕ=π for a defined λ, i.e., 532 nm, so the total efficiency is 1; hence this configuration requires a perfect PBS, zero absorbance by the active medium and a perfect angle θ. The other wavelengths will be affected by a different phase factor and the efficiency of the gate will suffer of an evident decay, as reported in Figure 10. In this sense the blue dashed curve can be thought as sort of transfer function that spectrally modulates a characteristic width for the signal passing through the gate.

**Figure 11:**
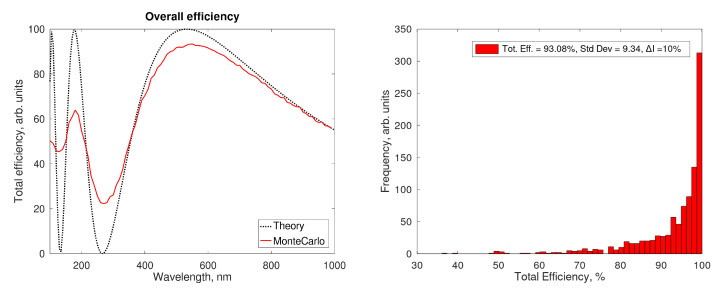
(**Left** panel) *Transfer function* of an ideal Kerr gate without (black dashed) and with (red solid) random fluctuations. We considered 1 mm of TiO${}_{2}$ as active medium, d=1μm, T=100 ps (E = 23.58 nJ; see Table 2) and the maximum fluctuations reported in Table 3, column “Errors”; (**Right** panel) total efficiency distribution obtained by a Monte Carlo simulation.

By introducing random fluctuations of the parameters involved (e.g., by a Monte Carlo model), in an ideal cell, the red curve in Figure 11 (left panel) is obtained. While the theoretical curve oscillates from 0% to 100% according to the phase variation of different wavelengths in the non-linear medium, the real total efficiency is restrained to the range 20–90%. In other words, the parameter fluctuations act as a smoothing filter on the transfer function in the wavelength domain.

The fluctuations will introduce a spread in the efficiency for ideal cells too: the distribution that can be obtained in this case is reported in Figure 11 (right panel). When fluctuations are uncorrelated, the optimal efficiency is often reached because of reciprocal compensation; the resulting broadening of the efficiency distribution is not dramatic.

### 3.7. Kerr-Gating: Conclusions

Picosecond time-domain Raman spectroscopy can be experimentally achieved with a Kerr gate, and the fluorescence rejection in measured Raman spectra could be obtained. For instance, a Kerr gate can reach response times as short as ≈3 ps if coupled with 10 mJ, 1 ps excitation pulses at 800 nm and a repetition frequency of 650 Hz [39]. However, optical transmittance of the Kerr gate in the open and closed states reaches low efficiencies because of to the incomplete polarization rotation in the Kerr medium and the losses in optical elements. In [17], the realization of an improved Kerr gate with optimized polarizers was reported. Operated with 1 ps pulse laser with at 1 kHz repetition rate, the device reached 4 ps of gating time, and the efficiency of the “open” state was improved to up to ≈40%, with an extinction ratio between the open and closed state of $10^{5}$. As a result of that efficient gating, the overall transmission and the collection efficiency of Raman light reached up to 1.6 times and 1.7 times, respectively, relative to their earlier results [39]. However, due to the limitations described so far, the Kerr gate method has not been widely used. The necessity of high-energy pulsed light, in fact, easily makes this method non-compliant with safety regulations, since photo-induced damage risk is increased [11].

## 4. Time-Gated Single Photon Avalanche Photodiode (TG-SPAD)

### 4.1. Introduction and Theory

SPADs have been extensively explored for the detection of low intensity light. The implementation of SPADs in complementary metal-oxide-semiconductor (CMOS) technologies has boosted the application of the CMOS SPADs, owing to their capability of being integrated with CMOS control electronics [40,41,42]. With the abilities of single photon detection and a sub-nanosecond temporal response, the CMOS SPAD has been used in various applications involving the time-correlated single photon counting (TCSPC) and time-gated detection applications, including fluorescence lifetime imaging (FLIM) [43,44], super-resolution FLIM [45], fluorescence fluctuation spectroscopy (FFS) [46], near infrared spectroscopy (NIRS) [47,48], light detection and ranging (LIDAR) and 3D time-of-flight [49,50,51], quantum mechanics [52], quantum communication [53] and quantum imaging [54,55]. A SPAD can be operated in either free running or time-gated (TG) modes. The fast gating capability makes the CMOS SPAD a low-cost alternative to ICCDs and a solution to be compared with an optical Kerr gate. Recently, CMOS TG-SPADs have been proposed for fluorescence rejection in Raman spectroscopy [56,57,58,59], where external pulse generators allowed one to control the detection system and sub-nanosecond windows were achieved.

**Figure 12:**
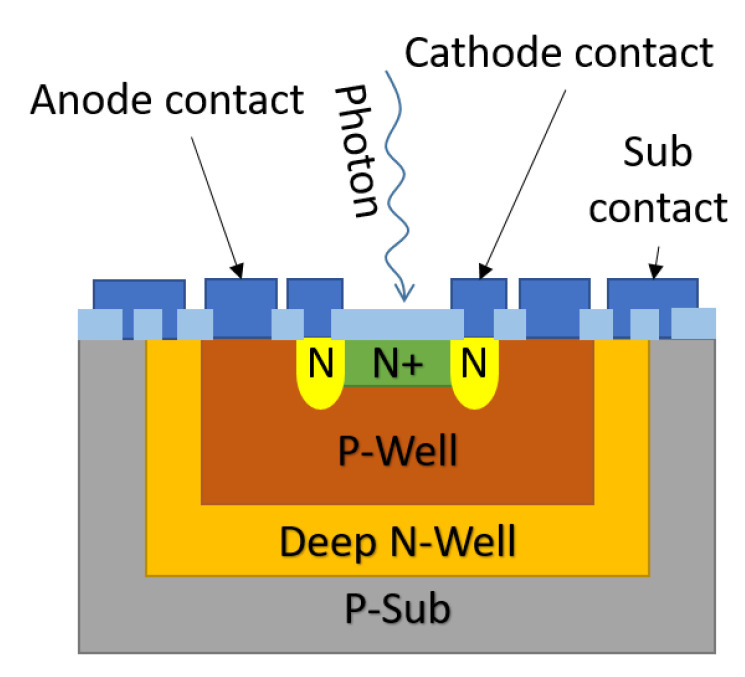
The junction scheme of a single photon avalanche photodiode (SPAD) device.

**Figure 13:**
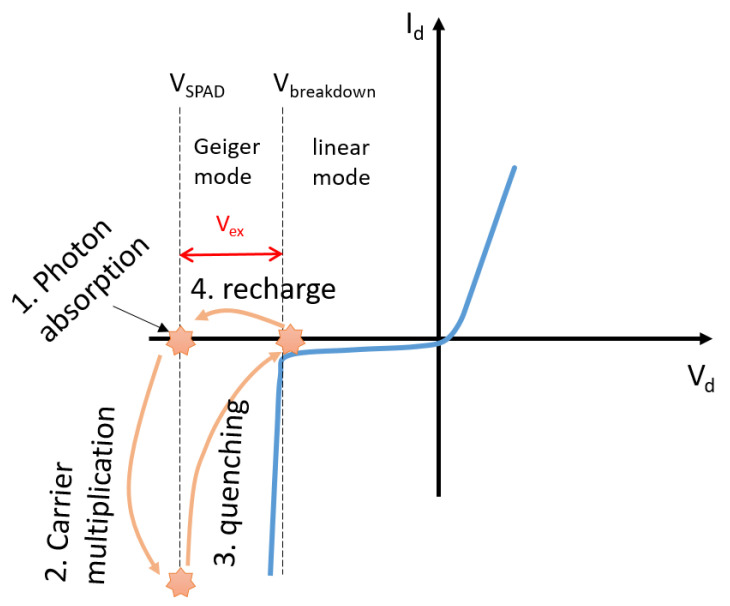
Current–voltage (IV) characteristic curve of an avalanche photodiode operated in Geiger mode.

A SPAD is essentially a p-n junction (see Figure 12) biased above the avalanche breakdown voltage (VBR), the so-called Geiger mode. Controlled by an external quenching circuit, the single photon detection process of the SPAD can be divided into four phases. These are carrier generation by a photon, internal carrier multiplication, quenching of the voltage across the SPAD and recharge. Figure 13 shows the four phases involved in a photon detection process on the I vs. V characteristic curve of the SPAD.

Before photon detection, the SPAD is biased above the VBR, and this overdrive voltage is named the excess bias ($V_{ex}$). The detection cycle of the SPAD begins when an absorbed photon results in the generation of an electron-hole pair of free carriers. In the second phase, the photon-generated carriers are accelerated by the high electric field and multiplied in the depletion region. As discussed in [60], basically, two processes are involved in this phase. The first process refers to impact ionization induced by the high electric field, which triggers the carrier multiplication process that creates an avalanche of secondary carriers. The ionization probability strongly depends on the electric field, which can be adjusted by applying different $V_{ex}$. The second process is internal quenching. The ionization process increases the local current density, which causes a larger voltage drop across the space charge resistor, and in turn weakens the ionization process. When the two processes are balanced, a current pulse in the mA range is then delivered to the external control circuit, which senses and quenches the avalanche.

The fast quenching of the avalanche process is important for the operation of the SPAD, because it protects the diode from overheating and related damages. After being fully quenched, the SPAD is recharged back to the Geiger mode. The entire detection cycle is shown in Figure 13. The external control circuit plays a dominant role in the detection cycle. The SPAD control circuit is designed to match a specific application. For instance, the SPAD can be designed in a pixel array or as a discrete component, operated in free running or time-gated mode. Each SPAD application has different requirements, and the quenching and recharge circuits of the SPAD must be designed accordingly.

The parameters commonly used to characterize the performance of a SPAD are described next.

1. *Dead time*: As depicted in Figure 13, during the time when a SPAD undergoes photon absorption, carrier multiplication, quenching and recharge, the SPAD is not able to detect a subsequent photon. Dead time is defined as the time it takes to complete a detection cycle, which determines the maximum counting rate of a SPAD. Therefore, fast quenching and recharge circuits are required for high-speed applications.
2. *Dark count rate* (DCR): DCR is defined as the number of counts per second when the SPAD is in the dark. A major source of DCR in a CMOS SPAD at room temperature is the thermal generation of free carriers, and the generation rate is also related to the ionization probability. Therefore, DCR depends on temperature, excess bias and the size of the active area of the SPAD. DCR determines the minimum incident photon rate that can be detected.
3. *Photon detection efficiency* (PDE): When a light source with certain intensity is incident on the detector, only a portion of the incident photons can be detected due to several reasons, including surface reflection, photon absorption before reaching the depletion region, absorption coefficient of the material and the probability of triggering an avalanche. PDE is defined as the ratio of the number of voltage pulses detected to the number of total incident photons. PDE is the most important parameter to evaluate the detection efficiency of a SPAD, and it is a function of the incident wavelength and excess bias. The PDE of a SPAD corresponds to the external quantum efficiency of other types of photodetectors.
4. *Afterpulsing probability* (AP): During the quenching phase, a large number of carriers flow through the depletion region. Some carriers are trapped in deep energy levels within the depletion region and released later to trigger a second detection cycle that is not initiated by photon absorption. The pulses generated by the released carriers from traps are named afterpulsing in SPAD. AP is correlated to the avalanche current and its duration, and thus, to the amount of charge of the avalanche pulse. Therefore, AP is proportional to the parasitic capacitance of the photodiode and the quenching time. The latter can be optimized by specially designed external control circuits.
5. *Fill factor* (FF): To achieve fast quenching and resetting, complex control circuits are designed for SPAD pixels. In active SPAD pixels, the area of the control circuits is often comparable to or larger than the optically active area of the SPAD. Fill factor is defined as the ratio of the SPAD active area to the total pixel area. For better usage of the chip area, a high FF is preferred.
6. *Timing skew* (TS): The shot noise of the events just described (i.e., parameters 1–5) contributes to distorting the spectra measured with time-resolved Raman spectrometers employing CMOS SPAD line sensors, and the so-called timing skew of the sensors [61,62,63]. Due to their spatial extension, a timing mismatch happens among the pixels of the sensor, i.e., the points of the spectrum. This leads in turn to shifted time gates for each pixel, so that photon counts at different spectral points may vary if the signal varies in time. This kind of sampling error can further distort the spectra.

DCR and PDE are strongly determined by the fabrication process, although some freedom is available for the designs of the control circuits in SPAD pixels. Optimized designs of quenching and recharge circuits mostly target reduction of the dead time and AP, and increase in FF. Two types of SPADs control circuits will be discussed: free running and time-gated.

### 4.2. Free Running Operation

“Passive quenching and recharge” should be the simplest approach for the design of a SPAD in free running mode. An example of a possible circuit to be used in this case was discussed in [64]. It consists of a quench resistor ($R_{Q}$) connected in series with the photodiode which is biased at ($V_{BR}$ + $V_{ex}$). To efficiently quench the avalanche current in a very short time, $R_{Q}$ is very large, usually in the range of kΩ to MΩ. Consequently, even a low avalanche current can result in a high enough voltage drop across $R_{Q}$. This voltage drop forces the bias of the SPAD to reduce below the breakdown voltage $V_{BR}$ and then quench the avalanche process. The time it takes to fully quench the avalanche current is determined by $R_{Q}$, and the dynamic resistance ($R_{D}$) and capacitance ($C_{D}$) of the diode.

**Figure 14:**
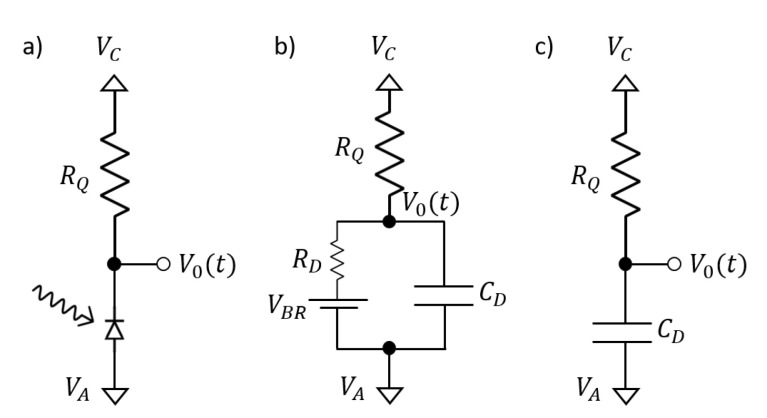
(**a**) A SPAD with passive quenching and recharge circuits; (**b**) equivalent circuit of passive quenching; (**c**) equivalent circuit of passive recharge.

Figure 14a shows a simple SPAD circuit with passive quenching and recharge [64]. The equivalent quenching and recharge circuits are given in Figure 14b,c respectively. The external biases are applied to the anode ($V_{A}$) and the quench resistor ($V_{C}$), and photon detection is sensed from the variations of the cathode potential [$V_{O}\left(t\right)$]. With this bias condition, the excess bias equals $V_{ex}=V_{C}-V_{A}-V_{BR}$.

By applying the Kirchhoff’s law to the passive quenching and recharge phases (Figure 14b,c), it is possible to estimate the time constants characterizing the processes:

(9) $$\tau_{quench}=\frac{C_{D}R_{D}R_{Q}}{R_{D}+R_{Q}},\frac{C_{D}R_{D}R_{Q}}{R_{D}+R_{Q}}=R_{D}\left|\right|R_{Q}$$

(10) $$\tau_{recharge}=C_{D}R_{Q}$$

The quench resistor ($R_{Q}$) dominates the recharge time, and the quenching time is correlated with the parallel combination of $R_{Q}$ and $R_{D}$. Since $R_{D}$ is much lower than $R_{Q}$, the quenching time is shorter than the recharge time: $10^{-1}$ ns vs. tens of nanoseconds.

In a passive quenching circuit, the quench resistor usually occupies an area smaller than the area of the photodiode, so a high FF can be achieved. Passive quenching is also suitable for the design of large pixel arrays. A drawback of the passive quenching circuit is the unstable dead time. If a photon arrives during the slow recharge phase, since the SPAD is biased at the Geiger mode, this photon could trigger a second detection and stop the recharge process. As a result, the dead time is expanded becoming longer than the designed one.

Another limitation of the passive quenching circuit is the AP. As mentioned above, AP is related to the quenching time and the parasitic capacitance. Since the circuit only contains a resistor and a photodiode, the circuit’s capacitance mainly comes from the capacitance of the photodiode. The capacitance of the photodiode is determined by the fabrication process, the SPAD area and reverse bias voltage. For SPADs implemented in deep sub-micron CMOS technologies, the capacitance is small and in the range of fF. This is an advantage of the passive quenching circuit, but the quenching time is long enough for carriers to be trapped. If the SPAD is in the Geiger mode, then trapped carriers can be released later, leading to afterpulsing. In addition, the captured carriers released during the long recharge phase can cause the dead time to increase. To solve the dead time and afterpulsing problems, high-speed control circuits are designed to reduce the quenching and reset times of the SPAD.

A possible improvement is represented by a mixed passive-active quenching circuit [65]. In this case, the dead time of the SPAD is effectively shortened.

Therefore, high counting rates can be achieved for SPADs with active quenching and recharge. As given in [66], a maximum counting rate of 185 MHz was achieved with a mixed passive-active quenching circuit, corresponding to a dead time of 540 ps. Regarding the AP, the parasitic capacitance at the node of the SPAD cathode was increased because of the complex connection, but the quenching time was also reduced. Further solutions were implemented in order to reduce the AP (e.g., to add a hold-off time). However, the FF of the mixed quenching circuit is low, since complicated control circuits are used. Hence, SPAD pixels with passive quenching and reset are still the preferred choice for design of large arrays.

The free running SPADs have been widely used in the time-resolved applications with the TCSPC technique, such as in FLIM and NIRS. Regarding Raman spectroscopy applications, the time-gated SPADs are more suitable due to its fluorescence suppression capability.

### 4.3. Time-Gated Operation

**Figure 15:**
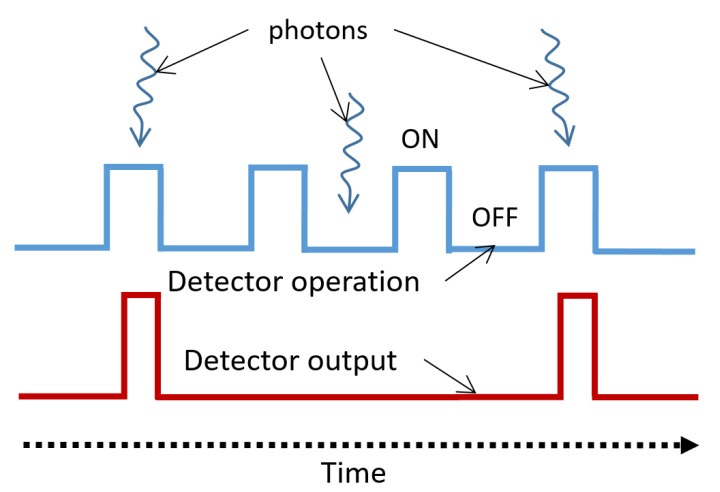
Time-gated operation.

The quenching and recharge circuits reviewed in the previous sub-section were mainly developed for free running SPADs [65,67]. However, there are applications in which the photon detection is required only for a short time window after a pulse excitation, and the time window has to be precisely synchronized with the excitation, as shown in Figure 15. This windowed mode of photodetection is known as the time-gated operation of SPAD. Typical examples requiring time-gated photodetection include Raman spectroscopy, to remove the fluorescence, and NIRS. To operate a SPAD in time-gated mode, the control circuit is crucial, because it quickly gates the SPAD above and below the avalanche breakdown voltage and synchronizes the detection with the pulse excitation.

**Figure 16:**
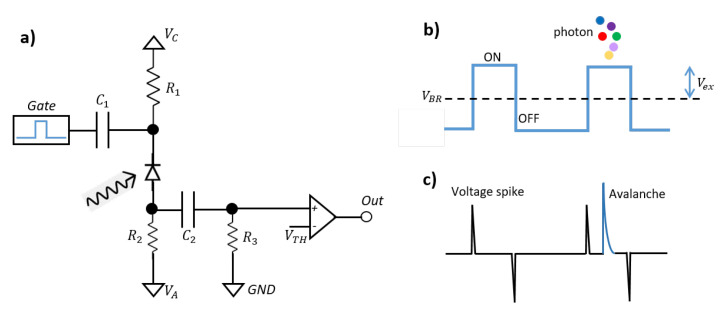
Time-gated operation scheme. (**a**) A basic diagram of a SPAD with a time gated control circuit. (**b**) A timing diagram of the gating signal; (**c**) a timing diagram of the output of the AC pick-up circuit.

In [64], a common way to apply the gating signal is described; see Figure 16a. A DC pre-bias is provided by a voltage source $V_{C}$ and the gating signal is coupled through the capacitor $C_{1}$. To achieve fast gating, $C_{1}$ should be small. However, to maintain long enough pulse duration, $C_{1}$ cannot be too small. Thus, the design of the input AC coupling network is important for appropriate gating and sensing of the SPAD.

To sense an avalanche occurring during the narrow gate, usually an AC pick-up circuit is applied and its output is fed to a high speed comparator.

Upon detection, the avalanche current flows through the resistors $R_{3}$ ($R_{2}>>R_{3}$), which quickly quenches the avalanche. Then, the SPAD is brought back to the Geiger mode by $R_{2}$. The function of the AC pick-up network is to sense the transient voltage increase of the SPAD’s anode that is induced by the avalanche current. However, because of the large $R_{2}$, a wide gate window is required, and this limits the SPAD’s maximum counting rate.

On the other hand, there are voltage spikes occurring during voltage transitions. As shown in Figure 16b, the SPAD is biased above and below the breakdown voltage. The fast voltage transition introduces a sudden variation to the anode voltage, and spikes (rising and falling) appear at the output of the pick-up network, as shown in Figure 16c. To reduce the effect of the spikes, the threshold ($V_{TH}$) of the comparator should be higher than their amplitude. Since the amplitude of the voltage spike is related to the transition speed and amplitude of the gating signal, the spike’s amplitude can be higher than the avalanche pulse. In these situations, a high value for $V_{TH}$ is not an efficient choice.

### 4.4. Raman

In this sub-section, several experimental approaches will be discussed in order to review the different experimental setups based on the SPADs that have been proposed in the literature and could be implemented for time-gated Raman spectroscopy.

Most of the papers proposed several versions of the same idea, which is to introduce arrays of SPADs characterized by an increasing size. This paper is not aimed at a detailed review of these systems, which has been recently published [68] (see Table 4 for an updated summary); hence we will focus only on the descriptions of some relevant examples. This information will be used to compare this approach with the others here presented.

Sensors based on a SPAD array were employed for imaging in several papers, and a relevant step towards in this field is represented by the experiment described in [69]; further examples are reported in [41,43,70]. A time-gated 128×128 CMOS SPAD array was triggered by a small fraction of the excitation laser; this optical trigger was detected by a single SPAD to convert the optical into an electrical signal. An external delay line was employed to synchronize the SPAD activation in order to optimize the measurement of the Raman spectrum on the sensor plane. This system was used to perform time gated FLIM and TRRS.

In [63] the spectrometer included a time-resolved, 16×256 line sensor, with an integrated 256-channel 3-bit on-chip time-to-digital converter (TDC). The SPAD array consisted of 16 columns and 256 rows. Each one of the 256 columns contained 16 SPADs and determined the signal in a single point of the Raman spectrum, providing a final spectrum of 256 points covering the wavenumber range of approximately 1600 cm${}^{-1}$. A suitable diffraction grating was employed to separate the different wavelengths with a final resolution of ≈6 cm${}^{-1}$. Excitation was provided by a 532 nm pulsed laser (pulse width: 160 ps; bandwidth: 0.11 nm; PRR: 350 kHz; maximum pulse energy: 1μJ). The SPAD was biased to the Geiger mode (i.e., single photon detection) by compensating the delay between the optical and electrical signals. This was achieved by sending a logic level trigger signal, generated by the optical detector, to the CMOS SPAD after the transition into an off-chip digital delay unit. The Geiger mode was activated only for short intervals; hence, the Raman signal was detected while the fluorescence photons were rejected. The arrival time of the photons was measured by an integrated on-chip 256-channel, 3-bit TDC with a resolution of ≈50 to ≈200 ps. The final time gate width was chosen in the data post-processing phase by selecting the analyzed time bins. The system was equipped also with a 3D mechanical stage to move the sample and a field-programmable gate array control circuit (FPGA) to drive the sensor circuit.

A fluorescence-suppressed Raman spectrometer can be employed for several applications. In [165] it was used to obtain the chemical imaging of human teeth. The experimental setup was based on a 16×256 time-resolved CMOS SPAD with an integrated 256-channel, 3-bit on-chip TDC converter [62,63]. A simple unsupervised machine learning algorithm, i.e., k-means clustering, was introduced to obtain high quality images which were compared with those achievable with commercial systems. The time-resolved spectra had a 4.4–8.8 times higher signal to peak-to-peak noise ratio by considering the same radiant exposure (≈300$\frac{J}{{\mathrm{mm}}^{2}}$) for both the spectrometers. The excitation source was a pulsed laser (wavelength: 532 nm; bandwidth: 0.11 nm; pulse width: 160 ps; pulse energy: 0.5 μJ; PRR: 350 kHz). Additionally, in this case, the SPAD was biased to the Geiger mode as described in the previous paragraph. Some of the laser pulses were sent to an optical detector in order to synchronize the Geiger mode with the excitation source. The detection setup was completed by a diffraction grating (final resolution: ≈6 cm${}^{-1}$) and an FPGA suitably programmed to drive the electronics involved.

In [140] the integrated TDC converters performed pre-calculations using using center-of-mass method (CMM). The sensor was characterized by a timing of 65,000 photons/pixel at 200 Hz line rate at 40 ps resolution. The array was composed by 256×2 SPADs and equipped with a TCSPC for each pixel which could generate histograms with 320 ps bin resolution. The array was characterized by two lines: the first one was optimized for detection of wavelength range 450–550 nm and named the blue line, and the second one was optimized for 600–900 nm and named the red line. The latter was designed to obtain higher detection efficiency and lower noise. In this work, two remarkable novelties were introduced: the pixels had individual TCSPC electronics and the spectrometer SPAD array had computational logic for the CMM calculation of photon arrival times on the fly. Parallelized TCSPC can be useful in low light circumstances. It can act as a measurement tool for fluorescence decays and time gating by post-processing the acquired TCSPC histograms. The CMM estimation improves photon detection efficiency, since in this case, it is not necessary to transfer TDC codes from the array to the related electronics.

Commercial TCSPC systems are usually single-channel systems. More complex devices, such as 8 channel [166] or 16 channel [167] TCSPC show several drawbacks since they are not fully independent. Indeed, at high count rates, the high probability of coincidence or cross-talk could have negative consequences on their performances.

In [168], a laser microchip pumped by 808 nm CW laser was employed (pulse duration: 100 ps; pulse energy at 1064 nm: >20 nJ; PRR: up to 500 kHz—variable according to the pump energy). As in most DPSS lasers, the narrow linewidth (<0.1 nm) was appropriate for Raman spectroscopy.

The pump was a diode laser, stabilized in wavelength (1064 nm), single-mode and fiber-coupled. A 40 μm FWHM Gaussian spot was focused on the laser microchip, and its output was collimated and sent through a miniaturized optical insulator to cut off back reflections. Light was then focused into a MgO:PPLN crystal, obtaining the harmonic at 532 nm with 60% conversion efficiency; collimated again; and the remaining 1064 nm light was removed using a short-pass filter. Significant drawbacks of this setup were the limited lifetime of the saturable absorber output coupler and the power of the pump laser. In fact, to minimize the risk of laser failure, the laser PRR was set significantly lower than its maximum capability.

The detector was a 1024×8 pixel SPAD array, well described in [56]. This SPAD array was designed and fabricated employing standard CMOS processes and features. The signal exiting the spectrometer was aligned so that about 90% fell in the two center rows of the SPAD, which actually operates as a 1024×2 array. Furthermore, the readout architecture was modified to operate the SPAD detector up to 1 MHz. A fast FPGA was used to accumulate the counts of each pixel over a large number of gates, and subsequently transfer these data at a slower rate to the computer. Excitation was provided by a 532 nm pulsed laser (pulse width: 400 ps; PRR: 1 kHz; pulse energy: 20 J/pulse). Due to the low laser repetition rate, the afterpulsing effect was negligible since the sensor had a dead time of 1 ms to release previously created trapped carriers. The laser light was used to trigger the detector, and a the optical signal was converted into an electrical one by exploiting a single pixel SPAD.

In [169] an optical spectrometer was built around a SPAD based 512 pixel line sensor. The excitation source uses a 532 nm pulsed laser diode, with a 4 kHz repetition rate, peak power of 6 kW, a pulse width of 0.6 ns and a spectral broadening line width narrower than 0.1 nm.

The CMOS sensor was a 16.5 giga-events/s 1024×8 SPAD line sensor with 23.78 μm pixel pitch. Every pixel has a 32 bin histogramming time-to-digital converter (TDC) with zoomable time ranges from 1.6 up to 204.8 ns. The sensor can operate in three main modalities:

1. Single photon counting (SPC) mode (with a throughput of 65 giga-events/s);
2. Time-correlated single photon counting (TCSPC) mode (194 million-events/s);
3. Histogramming mode (HistMode) (16.5 giga-events/s).

The histogramming mode generates on-chip TCSPC histograms at a per-pixel level to avoid a readout of raw TCSPC events, yielding up to two orders of increase in SPAD photon processing rates, enabling fast scanning or low-I/O-power, time-resolved spectroscopic imaging applications.

**Figure 17:**
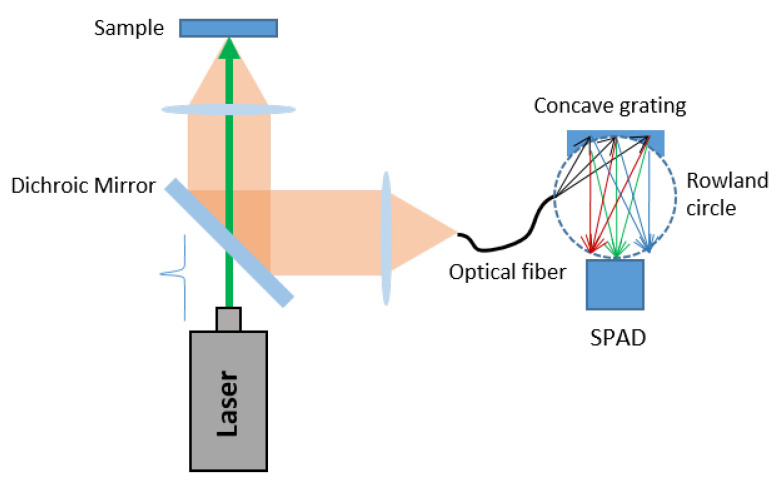
Optical diagram of the proposed TG Raman spectrometer reported in [59].

A completely different approach was introduced in [59,64], where a portable low-cost Raman spectrometer based on a concave grating and a single time-gated CMOS SPAD was designed. In this case the detector was moved and the measurement was not contextual for all the wavelengths; hence it takes longer than the setup previously described (see Figure 17 for the scheme of the proposed setup).

Unlike [56,58], in this case the SPAD was gated using on-chip pulse generators with a fixed gate window of 3.5 ns, which offered a minimum detection windows of ≈200 ps. The temporal resolution of the TG-SPAD was measured to be ≈60 ps with a very short laser pulse.

An optical fiber collects the signal towards the Rowland circle of the concave grating, as shown in Figure 17. In this configuration, the Rowland circle is defined as a circle characterized by a diameter equal to the distance between the grating and the SPAD surface which shares part of the circumference with the concave grating. By placing the entrance slit on the Rowland circle, different wavelengths will be focused in different points of the circumference. The whole spectrum can be acquired by moving a detector along the circle, but it is worth noting that focal plane of the Rowland circle is curved, while the active surface of the SPAD is planar. It follows that the whole SPAD area cannot be focused; hence the spectral resolution and the quality of the measurement are limited.

A 532 nm solid state laser was employed in this setup. The concave grating was optimized for the characterization of biological samples showing Raman shifts in the range of 500 cm${}^{-1}$ to 2000 cm${}^{-1}$ (i.e., from ≈545 to ≈595 nm).

Morimoto et al. in 2020 presented a 1 Mpixel SPAD camera [71] characterized by a 3.8 ns time gating and a 24 kfps frame rate. This sensor represents one of the most notable achievements regarding this research field. It was used to capture 2D/3D scenes over 2 m with resolution of 5.4 mm and precision better than 7.8 mm (rms). The camera was composed by two sections of 1024 × 500 pixels characterized by two different kinds of pixel.

The SPAD camera presented in [71] showed high performances also in complex imaging applications. In [164] it was employed to capture an ultrafast phenomena, such as the propagation of a light pulse. In this paper, the authors demonstrated that a video can be obtained by using a frame interval of 36 ps.

### 4.5. Interferometry: A Possible Resource

Interferometry could be employed to overcome the possible problems originating by the use of a SPAD array. A Fourier transform Raman spectrometer (FT-Raman) demonstrated to be a suitable approach allowing one to obtain the fluorescence rejection in Raman spectra by employing a near infrared (NIR) source (1064 nm) [170]. Nevertheless, in this case several drawbacks should be considered: NIR detectors have a remarkable cost and the Raman cross-section (i.e., $\lambda^{-4}$) suggests choosing a short excitation wavelength.

A further solution can be implemented by introducing a Fabry–Pérot interferometer (FPI) before the SPAD, allowing one to scan a wavelength range by moving only one of the two mirrors. Here an ideal configuration is considered: a FPI composed by two parallel partially reflective mirrors and an orthogonal monochromatic plane wave [171]. Real FPI are affected by several deficiencies [172] with consequences on the achievable spectral resolution.

**Figure 18:**
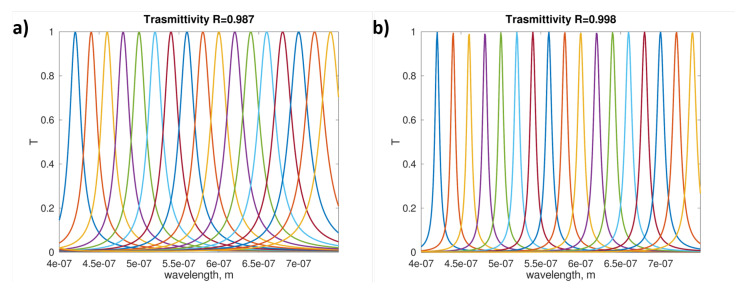
An ideal Fabry–Pérot interferometer (FPI) was considered; the first-order transmission maxima were obtained by scanning the distance *d* from 210 to 350 nm, with single steps equal to 10 nm. (**a**) The transmission profile of an ideal FPI composed by two infinite and identical extent plates (R=0.987), illuminated with a monochromatic plane wave at normal incidence; (**b**) the same simulation was performed with R=0.998.

The ideal resolution is determined only by the reflectivity of the mirrors, and thus, by the Finesse characterizing the designed optical cavity. In Figure 18 the results regarding two opposite configurations are reported. It can be straightforwardly concluded that it is possible to resolve the chosen 17 spectral elements in the visible range by suitably selecting the value of *R*.

In order to estimate the optimal setup to enable one to distinguish the 17 spectral elements previously introduced, a resolution power (RP) was defined. This choice was necessary because the curves reported in Figure 18a,b do not share the same width. This new parameter, which allows for a qualitative approach to the definition of the resolution capabilities of the designed setup, was evaluated as follows:

1. For each value of *R*, the FWHM (i.e., $width_{i}$) of the curves T vs. λ was suitably estimated.
2. If the sum of these values was higher (lower) than the wavelength interval which was considered, their ratio was higher (lower) than 1; i.e., $RP=\frac{\sum_{i}width_{i}}{\lambda_{max}-\lambda_{min}}>1$ (<1).

The results of further simulations are reported in Table 5. These can be used to estimate the necessary reflectivity *R* to be chosen in order to design a FPI able to resolve the desired wavelengths in the visible range. Two configurations were considered: a spectral resolution of 20 nm (2 nm) and 17 (165) spectral elements to be resolved. In the first case the threshold value R=0.994 represents a reflectivity achievable with low cost optical elements, while in the second one, unusually high-reflectivity mirrors could be employed and specific optical elements with R>0.99994 should be realized.

Furthermore, single steps of 10 nm can be realized with commercial solutions (e.g., piezo actuators) while smaller displacements can be affected by insufficient repeatability; it follows that this parameter assumes a fundamental role (to be carefully assessed) concerning the expected spectral resolution.

### 4.6. SPAD: Conclusions

SPAD imagers have not reached widespread use for far, mainly being research prototypes or spin-off of devices designed in academic environments. Moreover, large arrays (>256 channels) are not produced, so that for spectroscopic purposes they are not very competitive. Only small arrays can now be purchased (PhotonForce, a 32×32 time-resolved photon-counting camera [173]; MPD, with a 64×32 [174] camera). Other kinds of SPAD arrays on the market may either be derived from non-imaging silicon photo multipliers, used, for example, in clinical PET (Philips dSiPM); or from time-of-flight sensors (STMicroelectronics) [175]).

Beyond the experimental setups based on Kerr-gate, both PMTs [176,177] and ICCDs [178] have been employed in TGRS with sub-nanosecond gating. Though the latter kind of detectors are very sensitive, they are also much more expensive than SPADs; moreover, the electronic management of the devices is often embedded in the electronics and not completely customizable. The TG-SPAD-based CMOS pixel circuit can be used to reduce the cost and improve the integration of the detector. Nevertheless, it is worth noting that an array with few pixels, i.e., a few SPADs, could not guarantee the spectral resolution necessary for Raman spectroscopy. Moreover, due to the single-photon detection ability of the SPADs, it is not possible to acquire single-shot spectra, but accumulation of more laser shots is necessary, regardless of the single-pixel or array configuration.

## 5. Intensified CCD (ICCD)

### 5.1. Introduction and Theory

**Figure 19:**
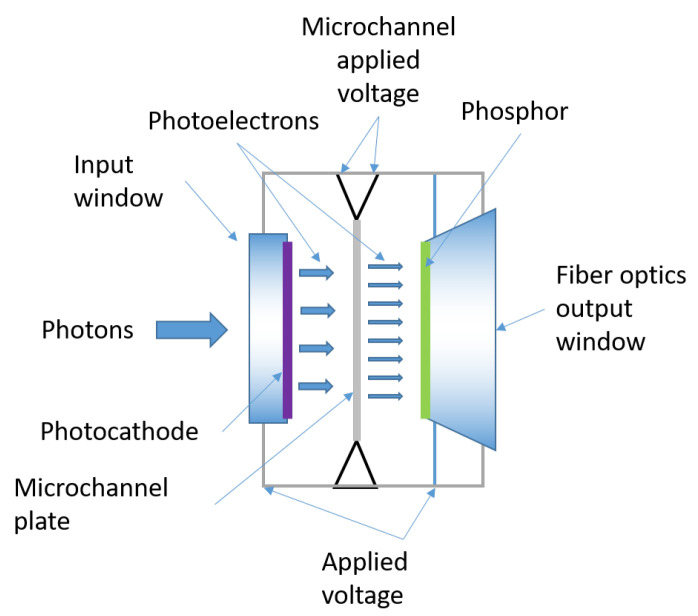
Intensified CCD (ICCD) scheme.

Intensified ICCDs are CCDs coupled to devices called image intensifiers, which allow photon multiplication (i.e., image intensification). The image intensifier is usually composed by three different elements: a photocathode, a micro-channel plate (MCP) and a phosphor screen (see Figure 19).

**Figure 20:**
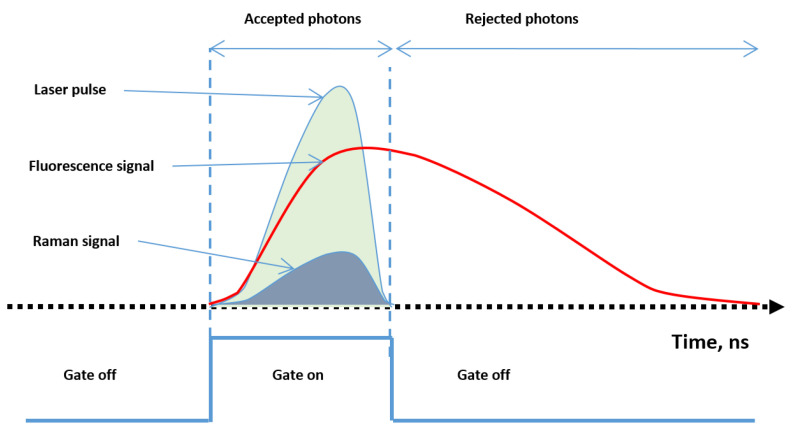
The time gating principle of ICCDs.

These elements are mounted in front of the CCD. Photons traveling towards the CCD hit the photocathode, generating photoelectrons. A control voltage, applied between the photocathode and MCP, accelerates these photoelectrons towards the MCP. This is composed by many thin glass capillaries (channels) whose internal walls are coated with a secondary electron emitting material. The electrons flying through the channels bump against the walls, and the repeated impacts cause them to multiply in number, so that the MCP acts as an electron multiplier device. The internal diameters of the channels range from 10 to 20 μm, bundled together to form a disk-shaped plate with a typical thickness of 0.5 to 1 mm. Through this process, each single electron can be multiplied into as many as 104 at the exit of the MCP. These are thereafter accelerated towards the phosphor screen, which finally converts the multiplied electrons back to photons, guided to the CCD by a fiber optic or a lens. Thus, the number of photons collected by the CCD sensor is always much larger than the number of photons originating from the dark current noise. For this reason, it is less necessary to cool an ICCD camera with respect to other systems, which is described in the following. The ability to switch on and off the MCP control voltage allows an ICCD to gather light down to very short time lapses (Figure 20) and then reject part of the fluorescence signal.

Previous intensifiers, named Gen II, were equipped with different types of photocathode materials, which allowed one to customize the image intensifier to the spectral requirements of the specific application. They were based on bi- or multi-alkali photocathodes. Indeed, the spectral response of an ICCD camera is primarily determined by the photocathode material used in the image intensifier.

Photocathodes of Gen II intensifiers used to provide quantum efficiencies of the order of only 25%. Gen III intensifiers, the third generation, also use the highly effective multi-channel plate, but in addition, they use semiconductor photocathodes (e.g., gallium arsenide photocathodes) that feature quantum efficiencies of more than 50%. These materials are typically doped to “tune” the response to particular wavelength ranges. (See Table 6 for examples of photocathodes mounted on commercial Andor ICCD).

The photocathodes can be applied to a quartz window that allows the photocathode response to extend into the UV range (≈180 nm). The quartz window can be substituted with a magnesium fluoride window to provide a response into the VUV (≈120 nm). If only a glass window is available, the spectral response is limited to wavelengths greater than the 350 nm.

Different photocathodes and their thicknesses can be “tuned” to optimize the wavelength response in different regions.

The primary impact of the intensifier is raising the signal-to-noise ratio, by amplifying the signal impacting the photocathode. On the other hand, in fact, the dark noise depends on the integration time and may derive from the thermal excitation of the photocathode and the CCD. The CCD can be cooled to reduce the dark current, while the photocathode cannot and produces a contribution that is amplified in the MCP. However, this is generally not an issue when using short gate times.

A second important effect of the presence of the intensifier is that it can be operated as a shutter: the emitted photoelectrons are accelerated towards the MCP only when a correct control voltage between the photocathode and the MCP is applied. When this voltage is reversed, the electrons return to the photocathode and there is no electron multiplication, nor is light emitted from the image intensifier. In this case the ICCD acts as a close shutter. This process, allowing image intensification only during the application of the control voltage at the photocathode is called gating. For this reason, ICCDs are also usually referred to as gateable CCDs.

Thus, besides the extremely high sensitivity of ICCD cameras, which enable single photon detection, the gateability is one of the major advantages of the ICCD. The best performing ICCD cameras enable shutter times as short as 200 ps (see Table 7 for a comparative report regarding three commercial products: Andor [179,180], Stanford [181,182] and Princeton [183]).

The ICCD can operate in the free running mode; in this case the camera is periodically gated by the built-in trigger source with different trigger frequencies and frame rates.

ICCDs are used in night vision devices and can be employed in various scientific applications, including time-resolved Raman spectroscopy [11,184]; depth analysis and profiling of multi-layer materials [178]; time-resolved fluorescence spectroscopy [185], quantum imaging [54]; fluorescence lifetime imaging [186]; and fluorescence detection and imaging [187].

An alternative to the ICCD is the electron-multiplying CCD (EMCCD), which contains an electron multiplying CCD sensor. In this case, the incoming photons are directly collected by the CCD sensor. The photo electrons generated by the sensor are then read out and afterwards multiplied electronically in a multi-stage gain register. For this reason, EMCCD cameras need extremely strong cooling (i.e., around 170 K), because the electrons originating from the dark current noise are of the same order of magnitude as the signal electrons under low-light conditions and are amplified together with them to the same extent. This powerful cooling system, however, contributes to raising the costs of an EMCCD and may yield condensation problems. Furthermore, EMCCDs do not provide as a fast gating capability as ICCD cameras do.

Anyway, the cost of EMCCDs is lower than ICCDs and and the resolution is usually better, since there is no need for the intensifier and larger sensors can be realized. Thus, EMCCDs may replace ICCDs in many applications that do not require time gating. Several studies were published regarding the comparison of ICCD/EMCCD/CCD and the errors related to each system [188,189].

A different method for achieving very high temporal resolution is based on the use of a streak camera [190,191]. A streak camera is a device in which:

1. Photons are converted into electrons by a photocathode.
2. Electrons are accelerated and deflected by a high speed sweep voltage.
3. Electrons are projected across a phosphor screen.
4. Electrons are converted back to photons by the phosphor screen.

**Figure 21:**
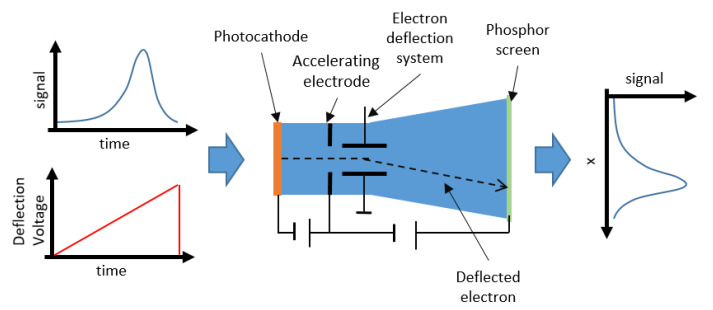
Working scheme of a streak camera. Through modulation of the electron deflection voltage, the deflection of the electrons emitted by the photocathode converts the time axis onto a spatial coordinate along the sweeping direction. If the entrance signal is a spectrum obtained by a dispersive medium, the streak camera allows one to resolve the intensity of the spectrum with time resolution as short as 200 fs.

The deflection system allows one to convert temporal variations of the entrance signal in spatial coordinate along the sweeping direction (Figure 21). If a time dependant 1D spectrum is projected in the streak camera, the output is a 2D image in which each line represents the spectrum at a different time. With such a system, temporal resolutions as short as 200 fs can be achieved. However, for Raman spectroscopy typical resolutions are of the order of tens of picoseconds.

### 5.2. State of the Art

In [11] it was proven that a picosecond excitation pulse coupled to a sub-nanosecond gating can lead to effective rejection of most fluorescence signal. This experiment represents a development of a previous work dealing with ps Raman spectroscopy using a streak camera [192] (10 ps time resolution, up to 2 kHz repetition rate) and a fluorophore with 4 ns of fluorescence lifetime.

It was possible to achieve a high level of background signal rejection both for solid and powder samples through a combination of an ordinary spectrograph coupled to an ultrafast, gated ICCD. A temporal resolution of about 150 ps was achieved, after 785 nm Ti:Sa laser excitation (PRR: 76 MHz; pulse duration: 2 ps; average power: 300 mW). It is worth noticing that the experiment was carried out without noticeable damage to the sample.

Since under a ps gating the number of Raman photons collected in one acquisition is usually very small, a high duty cycle was needed to obtain sufficient SNR. The image intensifier of the ICCD was able to work at very high repetition rate (up to 110 MHz) and very fast gating times (less than 200 ps, jitter lower than 20 ps). The photocathode employed was sensitive in the VIS-NIR (400–900 nm), and 65% quantum efficiency, and had a final resolution of 1376×1040 pixels.

The suitability of this approach for rejection of fluorescence was demonstrated in resonance Raman spectroscopy (RRS) by different groups [193]. Resonance in Raman spectra [8] happens when the incident photon energy is near an electronic transition of the investigated molecule: then the probability of transition is greatly enhanced and the intensity of Raman scattering, making it possible to study chemical compounds at very low concentrations.

In [193] a Raman instrument was assembled and tested that rejects typically 98–99% of background fluorescence. Use is made of ps laser pulses and an ultrafast ICCD for the time-gated detection in order to record the Raman signals during the pulse while blocking most of the fluorescence. The gated intensifier can be operated at 80 MHz, so high repetition rates and low pulse energies can be used, thereby minimizing photodegradation. For excitation, they use a frequency-tripled or doubled Ti:sapphire laser with a pulse width of 3 ps. It cannot be shorter because of the necessary spectral resolution for the RRS. The total instrumental resolution was 7 cm${}^{-1}$ in the the blue region and 15 cm${}^{-1}$ in the ultraviolet (UV) region. This setup allows one to use RRS for extra sensitivity and selectivity, even in the case of strong background fluorescence, and excitation wavelengths in the visible or UV range could be employed.

In [178] it was demonstrated that effective fluorescence rejection can be achieved using a low peak power, high repetition rate Ti:sapphire laser coupled to a fast-gated ICCD. This technical resource represents a useful tool in order to implement depth analysis and profiling of multi-layer materials with high performance, even in turbid conditions. In clear media, a confocal microscope works reasonably well to obtain depth resolution, although Raman signals originating from points above or below the focal point, though defocused, are collected and affect the depth resolution. In turbid media, both the excitation and Raman photons undergo multiple scattering events in all directions, so the focusing of deeper layers and the achievement of high depth resolution are usually problematic.

This approach seems to be promising with respect to the other ones reported in literature [194].

Time-gated Raman detection in depth profiling can be reached through time-gated acquisition after an appropriate delay; this allows one to discriminate between Raman photons originating from different layers. A laser excitation at 398 nm delivered 3 ps pulses at 76 MHz PRR. Tests over 1 and 2 mm of white powders and solid white polymers, respectively, allowed the evaluation of temporal discrimination and depth selectivity of the system on different combinations of first and second layers.

In [184], ICCDs were employed for standoff detection and identification of explosives and hazardous chemicals using TRRS. This system can be useful when it is not possible to employ the conventional ones based on Raman spectroscopy in a backscattering configuration, while employing a compact CCD-coupled spectrograph and a CW laser. Although these hand-held detectors can be made quite portable, they can be affected by a relevant drawback, since the very low signal could be dominated by the high fluorescence background from substrate or the sample itself. This signal can be suppressed using ICCDs since the time gating of these detectors can be synchronized with the laser pulse to allow data collection only in a short time after the Raman emission.

After rejecting the spurious light, the Raman signal from the sample is intensified by the ICCD. A transportable TGRS system was developed to detect and identify traces of explosives or derivatives on surfaces 10 m away from the sensor, and was tested in a simulated real field scenario. The system exploits as an excitation source the second harmonic of a Nd:YAG laser (wavelength: 532 nm; pulse duration: 4 ns; pulse energy: ≈100 mJ; PRR: 10 Hz). The signal is collected by an 8${}^{\prime \prime }$ telescope in monostatic geometry and sent to a Czerny turner spectrograph coupled to a gated ICCD. This geometry facilitates the focusing of the system at different distances to allow standoff identification from the suspected samples in an easier way. A variable time delay, whose width is of the same order of laser pulse duration, was imposed from the laser Q-switch trigger to the gate according to the distance from the target (i.e., 6.6 ns for each meter of distance). Good rejection of background light and fluorescence was achieved using a gate width of 10 ns (similar to the laser pulse duration). This timing capability, however, does not allow a very effective rejection of the short-lived fluorophores (of the order of ns).

A further example of setup designed for the standoff Raman spectroscopy is reported in [195]; here the tests were performed even on common materials involved in narcotics manufacture, and cocaine and heroin.

### 5.3. ICCD: Conclusions

Compared to the other approaches presented and discussed (i.e., the Kerr-gate and SPADs, offering ps and tens of a ps time resolution, respectively), at the current time, state-of-the-art ICCDs cannot reach the same temporal discrimination capabilities (the fastest ICCD show 200 ps as the shortest gating time). Nevertheless, for particular samples and applications (e.g., depth profiling or Raman analysis from standoff targets), the gated ICCDs shows good performances. On the other hand, for applications at the shortest distances, such as discrimination or selective probing of sub-millimetric layers, ICCDs are not fast enough, and setups involving both Kerr-gated or SPADs should be preferred. Moreover, the large dynamic range of CCDs allows single-shot collection of spectra, which is a key point in some applications, where the dose is limited by regulations or photo-damage of the sample.

Alternatives to CCDs could come from CMOS sensors. Although a CMOS camera offers advantages in cost, integration possibilities, power dissipation and system size when compared to a CCD, the latter offers lower noise and better SNR for photon detection up to the near-infrared region [196].

## 6. Summary and Conclusive Remarks

Weak Raman signals often suffer from overimposed fluorescence, both from the substrate and from the sample itself. In Section 1 we discussed the many approaches adopted to reduce the contribution of the fluoresce signal, whose fluctuations may completely mask the weaker Raman signal, aiming to weakening the fluorescence or to enhance the Raman instead. In this work we gave an overview of the specific, but relevant, sub-fields of time domain gating for cutting of the fluorescence signal, based on the different lifetimes of the two phenomena.

A theoretical derivation of performances achievable by time gated techniques in terms of signal-to-noise ratio was presented in Section 2. In Section 3, the properties of optical Kerr cell gating devices were investigated, and a MonteCarlo simulation of possible reductions in the efficiency of a Kerr gate by fluctuations of operational parameters (laser energy, optical inhomogeneity of the nonlinear medium, etc.) was discussed. Many realizations of Kerr cell-based gating devices were reported. In Section 4 the working principle and applications of single photon avalanche diodes were described, and in Section 5 the same was done with CCD sensors, both intensified by a microchannel plate or coupled with a streak camera.

In the end, these different approaches for fast time gating spectroscopy have been presented in this paper, and it was discussed how they differ in terms of performances, advantages, drawbacks and costs, as summarized in the following:

- Features
  1. Kerr-gate: the setup is based on a ps pulsed laser and a nonlinear medium acting as a switch when suitably activated. The detection should be implemented through a spectrometer coupled with a CCD or a CMOS.
  2. ICCD: The gating is fully accomplished by using the camera intensifier.
  3. TG-SPAD: The gating is realized by driving a SPAD with suitable electronics. Two different approaches can be followed: either a single SPAD spanning the wavelength range to be detected, or a SPAD array, measuring simultaneously all the wavelengths dispersed by a grating.
- Performances
  1. Kerr-gate: ps time-domain Raman spectroscopy can be achieved. The gating window has a similar duration as the employed laser pulse.
  2. ICCD: for particular samples and applications, the ICCD gate shows good performances. It is very sensitive and can improve the signal-to-noise ratio, allowing low-exposure spectroscopy.
  3. TG-SPAD: the temporal resolution can go from tens to hundreds of picoseconds and is related even to the pulse width of the laser.
- Drawbacks
  1. Kerr-gate: The gating and gated pulses must be accurately synchronized; the setup can reach remarkable size; high pulse energies are required; the activation time of the nonlinear medium can affect the theoretical performances achievable for a ps pulsed laser; the incomplete polarization rotation in the Kerr medium, the losses in optical elements and the optical transmittances of the Kerr gate do not allow one to obtain high efficiencies.
  2. ICCD: The performances are limited by the technology implemented in this kind of item. The temporal discrimination power of current ICCDs can only reach hundreds of picoseconds, making its exploitation and application limited when faster processes have to be investigated.
  3. TG-SPAD: Most SPAD imagers are still research prototypes, and only some characterized by limited size are commercially available.
- Cost
  It is high for all of the methods, though significant differences should be considered. In any case, a ps laser is necessary and can be expensive. Often it represents the main issue to be faced.
  1. Kerr-gate: the passive optical components that constitute the Kerr cell are relatively inexpensive.
  2. ICCD: the cost of the ICCD can surpass the cost of the laser, likely becoming the most expensive setup in the comparison.
  3. TG-SPAD: intermediate in cost compared to the previous solutions; it can be used to contain costs and improve the integration capability of the detection setup with the electronics of the developed device.

All the discussed methods can be used to extract the Raman signal when embedded with the fluorescence one, obtain a depth profiling or minimize the effect of photon scattering in thick samples and to reduce the statistical noise.

However, in addition to the presented methods, frequency-domain methods [197,198,199], wavelength-domain methods [200,201,202,203] and computational methods [204,205,206,207,208,209,210,211,212,213] represent further approaches that could be implemented, although with lower performances [3].

## Figures and Tables

**Table 1:** Coefficients between pulse length full width half maximum (FWHM) and best gating time for some values of a. The values represent the angular coefficients of the linear regression between the best gating time and the laser pulse width FWHM. To evaluate the goodness of linear fit, the $r^{2}$ coefficients are also shown.

Asymmetry Factor α	0	0.2	0.4	0.6	0.8
Best gating/FWHM	1.72	1.68	1.66	1.65	1.65
$r^{2}$	0.99922	0.99881	0.99801	0.99734	0.99723

**Table 2:** Pulse energy (J) to obtain ϕ=π in a Kerr cell (active medium: 1 mm of TiO${}_{2}$) at 532 nm.

	d [μm]	1	10	100	1000
T [ps]					
1		$2.358\times 10^{-10}$	$2.358\times 10^{-8}$	$2.358\times 10^{-6}$	$2.358\times 10^{-4}$
10		$2.358\times 10^{-9}$	$2.358\times 10^{-7}$	$2.358\times 10^{-5}$	$2.358\times 10^{-3}$
100		$2.358\times 10^{-8}$	$2.358\times 10^{-6}$	$2.358\times 10^{-4}$	$2.358\times 10^{-2}$

**Table 3:** Values considered for the parameters and their uncertainties.

Parameter	Symbol	Value	Uncertainty	Notes
refractive index	n	2.6		
non-linear refractive index	$n_{2}$	$8.86\times 10^{-19}$ m${}^{2}$/W	10%	underestimation
non-linear medium thickness	*L*	$10^{-3}$ m	$10^{-4}$ m	underestimation
wavelength	λ	$5.32\times 10^{-7}$ m	$6\times 10^{-10}$ m	laser line broadening
beam diameter in non-linear medium	*d*	$1.3\times 10^{-6}$ m	NO	from beam shaper datasheet
pulse duration	*T*	$88\times 10^{-12}$ s	NO	—
average power	*W*	0.34 W	NO	—
pulse repetition rate	PRR	$8\times 10^{7}$ Hz	NO	80 MHz (from laser datasheet)
average power density	*I*	estimated from W, PRR, T, d	10–50%	—

**Table 4:** SPAD: state of the art, according to [56,59,68,71]. The column *Technology* reports a number in (nm), and acronyms refer to specific semiconductor manufacturing processes and design rules employed to fabricate each SPAD. HV: high-voltage process; CIS: CMOS imaging sensor process; 3D: 3D integration technology (usually backside illuminated—BSI).

Sensor	Year	SPAD Array	Technology [nm]	Pixel Pitch [μm]	Fill Factor [%]	PDE [%]	DCR [cps/μm${}^{2}$]
[72]	2003	8 × 4	800	–	<1	0.2	1.6
[73,74,75,76,77,78,79]	2007	8 × 1	–	198	5	2.5	1.0
[80,81]	2007	64 × 64	350/HV	40	<1	0.1	71.0
[82,83,84]	2008	128 × 128	350/HV	25	–	–	17.0
[85,86]	2009	4 × 4	350/HV	36	<1	–	1.0
[87,88,89]	2009	60 × 48	350/HV	85	<1	0.1	7.0
[90,91,92,93]	2009	32 × 32	350/HV	100	3.1	1.3	12.7
[43,94,95,96,97,98,99,100,101,102,103,104]	2009	32 × 32	130/CIS	50	1	0.4	4.0
[44]	2009	64 × 4	350/HV	26	34	10.9	4.6
[105,106,107,108]	2009	32 × 32	350/HV	30	3.1	1.1	5.0
[109]	2009	7 × 2	350/HV	–	–	–	13.0
[110,111]	2011	128 × 128	350/HV	25	–	–	6.6
[112,113]	2011	160 × 128	130/CIS	50	1	0.3	2.0
[114,115,116]	2011	32 × 32	350/HV	25	20.3	–	5.4
[117,118]	2012	32 × 32	350/3D	50	75.4	–	39.7
[119,120,121]	2012	32 × 32	130/CIS	22	10	–	13.7
[122,123]	2013	64 × 64	130	48	<1	0.3	28.0
[124]	2013	416 × 4 × 4	350/HV	30×50	55.6	17.0	39.0
[56,125]	2013	1024 × 8	350/HV	24	44.3	9.6	29.0
[57,58,126,127,128]	2013	128 × 8	350/HV	33	23	5.8	71.0
[129,130,131,132]	2013	720 × 16 × 8	130/CIS	19	42.9	12.0	6.2
[70]	2013	10 × 43	350/HV	20×100	67	4	–
[133,134,135,136,137,138]	2014	512 × 128	350/HV	24	–	–	12.0
[59,64]	2014	1	130	10	9.8	3	–
[51]	2014	32 × 32	350	150	3.14	20–55%	120 cps
[139]	2015	416 × 18 × 9	350/HV	30×50	57	18.6	43.0
[140,141]	2015	256 × 2	130/CIS	24	43.7	–	5.4
[142]	2015	256 × 256	130/CIS	8	19.6	–	4.0
[143,144]	2015	400 × 1	130/3D	11	23.3	2.8	357.0
[145]	2016	128 × 120	65/3D	8	45	12.4	36.2
[146]	2016	72 × 60	180	35	14.4	0.4	2.3
[147,148]	2016	256 × 1	350/HV	24	40	13.6	11.0
[149]	2016	160 × 120	350/HV	15	21	–	12.0
[150,151,152]	2016	320 × 240	130/CIS	8/16	–	–	3.0
[153]	2017	1024 × 16	130/CIS	24	49.3	–	–
[61,62]	2017	256 × 16	350/HV	35	26	–	–
[154]	2017	256 × 8	130/CIS	24	43.7	–	5.4
[50,155,156,157]	2017	32 × 32	180	28	28	13.4	0.6
[158,159,160]	2017	512 × 512	180	16	10.5	5.2	0.3
[161]	2018	256 × 256	130/CIS	16	61	–	51.0
[162]	2019	400 × 400	65/CIS	6	70	–	100 cps
[163]	2019	256 × 256	40/90/3D	9.2	51.0	11.7	20 cps
[71,164]	2020	1024 × 1000	180	9.4	7.0/13.4	0.7/3.6	0.4/2.0 cps

**Table 5:** Coefficient obtained with the polynomial fit resolution power (RP) vs. reflectivity (*R*): $y=a\cdot x^{2}+b\cdot x+c$.

Resolution [nm]	*a*	*b*	*c*	RP = 1
20	−3002.6	5881.7	−2878.7	*R* = 0.994
2	−15,916,262.3	31,822,936.8	−15,906,674	*R* = 0.99994

**Table 6:** Photocathodes mounted on the commercial Andor ICCD.

Photocathode	Type	Peak QE	Min. Gating Speed
1	Gen 2	18%	<2 ns
2	Gen 2	16%	<5 ns
3	Gen 2	13.5%	<50 ns
4	Gen 3	48%	<2 ns
5	Gen 3	26%	<2 ns
6	Gen 2	25%	<100 ns
7	Gen 3	4%	<3 ns
8	Gen 3	40%	<2 ns
9	Gen 2	22%	<2 ns

**Table 7:** Comparative analysis of the main commercial items: Andor [179,180], Stanford [181,182] and Princeton [183].

	MODELS		
	iStar CCD 320, 334, 340, sCMOS	Stanford High Resolution: ns, ps	PI-MAX4:1024i
**Pixel Matrix**	1024 × 256, 1024 × 1024, 2048 × 512, 2560 × 2160	1360 × 1024	1024 × 1024
**Pixel Size** **[μm]**	26, 13, 13.5, 6.5	4.7	12.8
**Min. exposure time**	2 ns	1.2 ns (down to 200 ps)	2 ns (down to 200 ps)
**Max. Frame rate (fps)**	15.9, 4.2, 2.5, 50	14.0 (12/14 bit mode), 17.5 (8 bit mode)	26
**Optical shutter repetition rate** (Maximum photocathode repetition rate)	standard: 5 and 500 kHz, 3.3 MHz (burst mode)	standard: 200 kHz, 3.3 MHz (burst mode)	1 MHz; 100 kHz with ps gating
**Active Pixels**	18 mm tube 690 × 255 pixels 18 × 6.6 mm (25 mm tube 960 × 255 pixels 25 × 6.6 mm) 18 mm tube 1024 × 1024 pixels 13.3 × 13.3 mm 18 mm tube 1330 × 512 pixels 18 × 6.9 mm (25 mm tube 1850 × 512 pixels 25 × 6.9 mm) 18 mm tube 2554 × 2154 16.6 × 14.0 mm (25 mm tube 2560 × 2160 16.6 × 14.0 mm)	18 mm: 14.4 × 10.8 mm	13.1 × 13.1 mm
**Dynamic data range**	12-bit and 16-bit	14-bit up to 21-bit	16 bit
**CCD output**	Up to 16 bit, Up to 32 bit	standard: 12 bit—optional: 14 bit	16 bit
**Jitter**	<0.035 ns	<0.02 ns, <0.01 ns	<0.035 ns
**Gain**	Depends on several elements (e.g., phosphor screen, intensifiers)		
**Phosphor screen**	standard: P43, optional: P46		standard: P43, optional: P46, P47
